# Pancreas agenesis mutations disrupt a lead enhancer controlling a developmental enhancer cluster

**DOI:** 10.1016/j.devcel.2022.07.014

**Published:** 2022-08-22

**Authors:** Irene Miguel-Escalada, Miguel Ángel Maestro, Diego Balboa, Anamaria Elek, Aina Bernal, Edgar Bernardo, Vanessa Grau, Javier García-Hurtado, Arnau Sebé-Pedrós, Jorge Ferrer

**Affiliations:** 1 Centre for Genomic Regulation (CRG), The Barcelona Institute of Science and Technology, Dr. Aiguader 88, Barcelona 08003, Spain; 2 CIBER de Diabetes y Enfermedades Metabólicas Asociadas, Instituto de Salud Carlos III, Spain; 3 Genetics and Genomics Section, Department of Metabolism, Digestion and Reproduction, National Institute for Health Research (NIHR) Imperial Biomedical Research Centre, Imperial College London, London W12 0NN, UK; 4 Universitat Pompeu Fabra (UPF), Barcelona 08003, Spain

**Keywords:** enhancers, non-coding mutations, Mendelian disease, diabetes mellitus, stem cell differentiation, PTF1A, NEUROG3, endocrine differentiation, pancreas development

## Abstract

Sequence variants in *cis*-acting enhancers are important for polygenic disease, but their role in Mendelian disease is poorly understood. Redundancy between enhancers that regulate the same gene is thought to mitigate the pathogenic impact of enhancer mutations. Recent findings, however, have shown that loss-of-function mutations in a single enhancer near *PTF1A* cause pancreas agenesis and neonatal diabetes. Using mouse and human genetic models, we show that this enhancer activates an entire *PTF1A* enhancer cluster in early pancreatic multipotent progenitors. This leading role, therefore, precludes functional redundancy. We further demonstrate that transient expression of *PTF1A* in multipotent progenitors sets in motion an epigenetic cascade that is required for duct and endocrine differentiation. These findings shed insights into the genome regulatory mechanisms that drive pancreas differentiation. Furthermore, they reveal an enhancer that acts as a regulatory master key and is thus vulnerable to pathogenic loss-of-function mutations.

## Highlights

• The pancreas agenesis enhancer (EnhP) activates *PTF1A* in early pancreatic progenitors
• EnhP also activates other progenitor *PTF1A* enhancers
• This master key function explains why EnhP is vulnerable to loss-of-function mutations
• Transient PTF1A expression in progenitors controls pancreas growth and endocrinogenesis

## Graphical abstract

**Figure:**
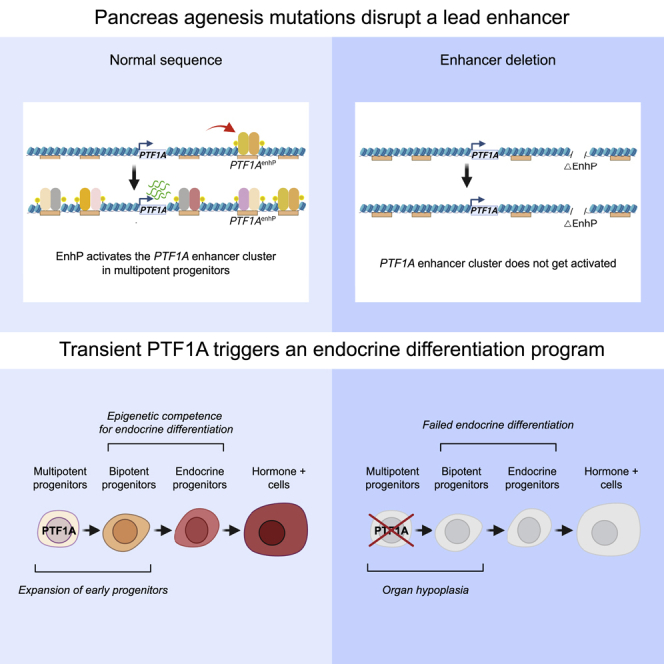

## Teaser

Miguel-Escalada et al. show that pancreas agenesis mutations disrupt a lead enhancer that activates other *PTF1A* enhancers in early progenitors of the embryonic pancreas. This enables transient PTF1A expression in progenitors, which controls pancreas growth and creates epigenetic competence for subsequent endocrine differentiation.

## Introduction

Most known Mendelian defects alter protein-coding sequences, although a sizable fraction of patients with suspected monogenic disease do not have a recognized genetic cause (Chong et al., 2015). In many such patients, the causal mutations could reside in non-coding DNA sequences, particularly in transcriptional enhancers that control the spatiotemporal regulation of genes. The advent of clinical whole genome sequencing together with the availability of enhancer maps promises to uncover many more pathogenic enhancer mutations. There are, however, hundreds of thousands of human enhancers with millions of sequence variants, and our understanding of which could be deleterious is extremely limited.

A limited number of monogenic disorders has already been shown to be caused by non-coding mutations (Gordon and Lyonnet, 2014; Miguel-Escalada et al., 2015; Spielmann et al., 2018). Several examples are caused by large structural variants that delete multiple enhancers (Benko et al., 2009; Long et al., 2020; Loots et al., 2005), as well as duplications and rearrangements that modify regulatory landscapes, causing gene silencing or misexpression (Kurth et al., 2009; Laugsch et al., 2019; Lupiáñez et al., 2015; Ngcungcu et al., 2017). Misexpression can also result from gain-of-function point mutations in distal enhancers, as shown for polydactyly (Lettice et al., 2012). The pathogenic role of loss-of-function mutations in single enhancers is less established. Enhancers form multi-enhancer clusters that regulate one or more genes, and experimental studies have shown that the loss of single enhancers is generally buffered by functionally redundant (“shadow”) enhancers, which in turn has led to a widely held concept that this genetic mechanism is unlikely to play a significant role in monogenic disease (Cannavò et al., 2016; Hay et al., 2016; Kvon et al., 2021; Osterwalder et al., 2018; Spielmann et al., 2018). However, it remains plausible that not all predicted enhancers are functionally equivalent and that some functions are indeed sensitive to loss-of-function mutations.

Isolated pancreatic agenesis (PAGEN2, OMIM 615935) provides a remarkable paradigm to understand why specific enhancers could be vulnerable to loss of function. Recessive loss-of-function mutations in a putative enhancer located ∼25 kb from *PTF1A* cause isolated pancreatic agenesis, which results in neonatal diabetes and exocrine insufficiency (Weedon et al., 2014). More than 40 probands have been found to carry at least 6 different point mutations in a single putative enhancer, including some that disrupt binding by pancreatic transcription factors PDX1 or FOXA2, or a deletion of the entire enhancer (Evliyaoğlu et al., 2018; Gabbay et al., 2017; Gonc et al., 2015; Weedon et al., 2014). Homozygous truncating mutations in the nearby gene *PTF1A*, encoding a basic helix-loop-helix transcription factor, also cause pancreas agenesis, as well as cerebellar agenesis (Sellick et al., 2004). Likewise, *Ptf1a* null mutant mice are born with a negligible pancreas structure (Kawaguchi et al., 2002; Krapp et al., 1998) as well as spinal cord, cerebellar, and retinal defects (Glasgow et al., 2005; Krapp et al., 1998; Pascual et al., 2007). Thus, pancreas agenesis enhancer mutations lead to a spatially restricted loss of *PTF1A* function in the pancreas. Although examples of putative loss-of-function enhancer mutations at other loci have also been described in individual cases or families (Benko et al., 2009, 2011; Bhatia et al., 2013; Ghiasvand et al., 2011; Smemo et al., 2012), pancreas agenesis provides a compelling demonstration of this genetic mechanism.

In this study, we have created mouse and human models that disrupt the enhancer that is mutated in pancreas agenesis (hereafter *PTF1A*^enhP^). This showed that *PTF1A*^enhP^ selectively regulates transient expression of *PTF1A* in the multipotent progenitor cells (MPCs) that form early embryonic pancreatic buds. *PTF1A*^enhP^ mutants did not completely block pancreas development, which allowed to analyze the function of PTF1A in pancreatic MPCs. Using stage-specific single-cell chromatin profiles, we show that PTF1A orchestrates an epigenetic endocrine differentiation program. We also demonstrate that *PTF1A*^enhP^ has a distinct enhancer function: it enables the activation of the entire *PTF1A* locus enhancer cluster. This suggests that a subset of enhancers can act as master keys to activate enhancer clusters, and solves a paradox of how mutations in a single enhancer can be catastrophic despite the existence of multiple other enhancers regulating the same gene.

## Results

### Pancreatic hypoplasia and diabetes in mice with enhancer deletions

**Figure 1:**
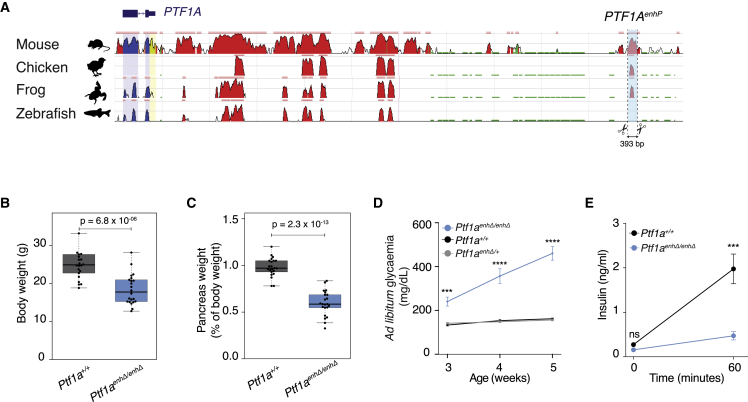
*Ptf1a* enhancer deletion in mice causes pancreatic hypoplasia and diabetes (A) Human *PTF1A* locus and ECR Browser conservation tracks. Sequences with >70% similarity over 100 bp in pairwise alignments are identified by horizontal pink lines on top of each track. The location of the mouse *Ptf1a*^enhP^ deletion is shown below. (B and C) (B) Body weight and (C) pancreas weight (expressed as percentage of body weight) in 6- to 11-week-old mice (n = 22 each genotype, Student’s t test p values). (D) *Ad libitum* glycemia of male mice after weaning (n = 22 each genotype). (E) Basal and post-fed plasma insulin from 7-week-old male mice (n = 7 each genotype) after an overnight fast. (D) and (E) show means ± SEM. Student’s t test. ^∗∗∗^p ≤ 0.0001, ^∗∗∗∗^p ≤ 0.00001. See also Figure S1.

The *PTF1A*^enhP^ sequence that is mutated in neonatal diabetes is highly conserved in human, mouse, and other tetrapod genomes (Figure 1A). To model the consequences of disrupting this enhancer, we used CRISPR-Cas9 to generate mice carrying a 393-bp deletion that spans all known human point mutations (Figures 1A and S1A–S1C) (Gabbay et al., 2017; Weedon et al., 2014). Mice carrying homozygous deletions (*Ptf1a*^*enhΔ/enhΔ*^) were born at the expected Mendelian ratios but were smaller than wild-type littermates (*Ptf1a*^*+/+*^; Figure 1B) and exhibited pancreatic hypoplasia—even after correcting for the reduced body weight, their median pancreas weight was ∼60% of their wild-type littermates (Figures 1C and S1D). Reduced body weight is consistent with intrauterine growth restriction seen patients with *PTF1A*^enhP^ mutations (Demirbilek et al., 2020). Hypoplasia was consistent with the phenotypic spectrum observed in patients with *PTF1A*^enhP^ mutations, which ranges from no visible pancreas to a reduced pancreas size in imaging studies, although some patients develop diabetes or exocrine insufficiency several years after birth (Demirbilek et al., 2020; Gabbay et al., 2017; Gonc et al., 2015; Weedon et al., 2014). *Ptf1a*^*enhΔ/enhΔ*^ mice were severely hyperglycemic soon after birth (Figure 1D) and showed decreased fed insulin (average 1.98 versus 0.34 ng/mL in *Ptf1a*^*+/+*^ versus *Ptf1a*^*enhΔ/enhΔ*^, respectively, p < 1 × 10^−04^; Figure 1E). Thus, homozygous deletion of *PTF1A*^enhP^ in mice led to pancreatic hypoplasia and insulin-deficient neonatal diabetes. The mouse phenotype therefore recapitulated salient features of the human mutations affecting this enhancer.

### *PTF1A*^enhP^ controls *PTF1A* selectively in pancreatic multipotent progenitors

**Figure 2:**
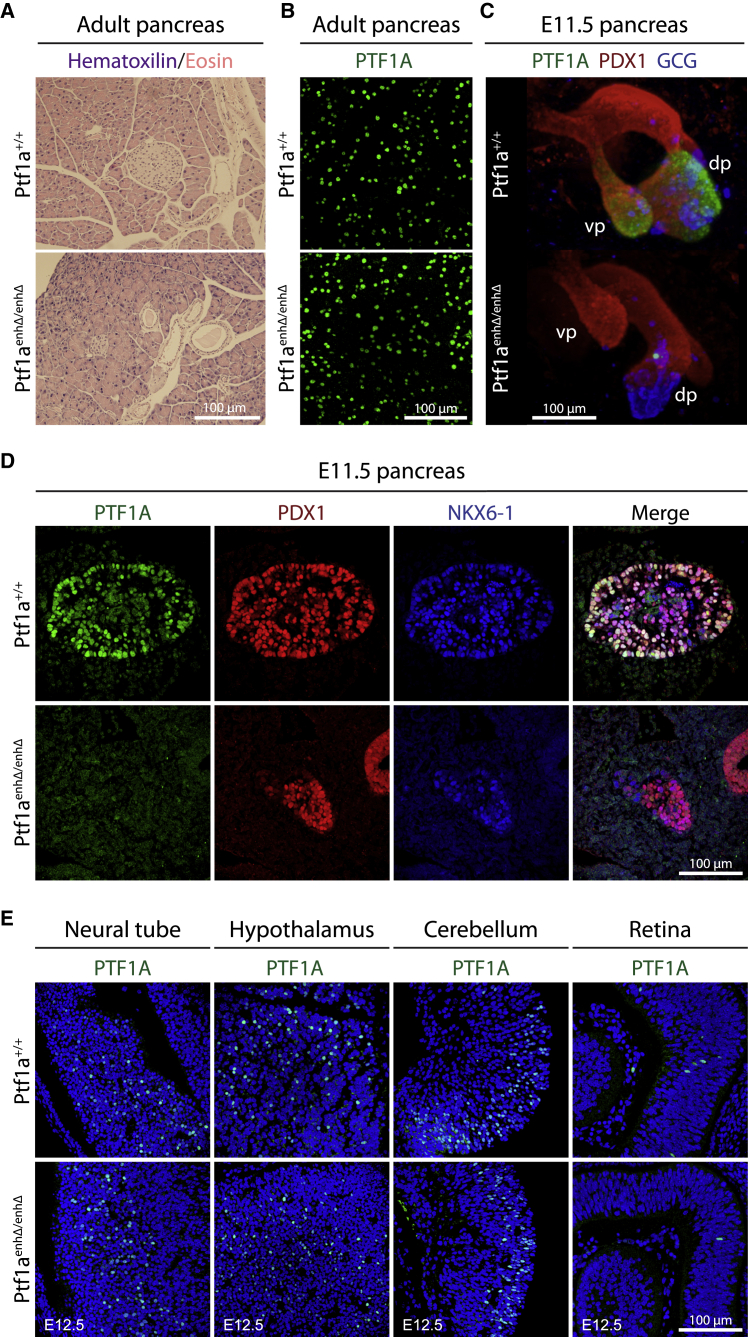
*Ptf1a*^*enhP*^ controls *Ptf1a* expression in mouse multipotent pancreatic progenitors (A) HE staining of pancreas from adult control and *Ptf1a*^*enhΔ/enhΔ*^ mice. (B) PTF1A immunofluorescence was preserved in acinar cells from adult *Ptf1a*^*enhΔ/enhΔ*^ pancreas. (C) 3D reconstructions of E11.5 pancreatic buds from *in toto* immunofluorescence stainings for PTF1A (green), PDX1 (red), and glucagon (GCG, blue). See also Video S1. (D) PTF1A (green) was depleted in dorsal pancreas from E11.5 *Ptf1a*^*enhΔ/enhΔ*^ embryos. PDX1 (red) and NKX6.1 (blue) were co-stained to label MPCs. (E) PTF1A expression in sagittal sections from control and mutant E12.5 neural tube, hypothalamus, cerebellum, and retinal cells. See also Figure S1.

Despite the marked reduction of pancreas size in postnatal *Ptf1a*^*enhΔ/enhΔ*^ mice, the morphology of acini, islets, and ducts in the remnant organ was largely conserved, with moderate dilation of large ducts (Figure 2A). PTF1A was first recognized as a pancreatic exocrine acinar cell-enriched transcription factor (Krapp et al., 1996, 1998); however, unexpectedly, mice with homozygous *PTF1A* enhancer deletions showed normal PTF1A expression in acinar cells (Figure 2B). *PTF1A*, however, is also transiently expressed at low levels in the MPCs that form early embryonic pancreatic buds and later give rise to all pancreatic epithelial lineages (Kawaguchi et al., 2002). We performed 3D reconstructions from *in toto*-stained E11.5 embryos, which showed that ventral and dorsal buds were formed in *Ptf1a*^*enhΔ/enhΔ*^ embryos, with a slight reduction in size of the dorsal bud and a wider stalk (Figure 2C; Video S1). The MPCs that formed such buds in *Ptf1a*^*enhΔ/enhΔ*^ embryos did not express PTF1A (Figures 2C, 2D, and S1E–S1H; Video S1). These results therefore indicate that *PTF1A*^enhP^ is not required for the specification of MPCs and formation of mouse pancreatic buds, but it is essential for *Ptf1a* expression at this stage and dispensable in the pancreatic acinar lineage.

Video S1. Lack of PTF1A expression and moderate hypoplasia of mutant pancreatic buds Selective Plane Illumination Microscopy was used for 3D reconstructions of *in toto*-stained embryonic pancreas from E11.5 *Ptf1a*^*+/+*^ (top) and *Ptf1a*^*enhΔ/enhΔ*^ (bottom) embryos. Pancreatic buds were stained for PDX1 (red), PTF1A (green) and glucagon (blue), related to Figure 2C.

PTF1A also plays critical roles in programming neuronal lineages and is expressed in precursors of the cerebellum (Hoshino et al., 2005), spinal cord (Glasgow et al., 2005), and retina (Fujitani et al., 2006). *Ptf1a*^*enhΔ/enhΔ*^ embryos, however, showed normal PTF1A expression in E11.5–E13.5 neural tube, as well as E12.5–E13.5 hypothalamus, cerebellum, and retina, although there was a reduced number of retinal PTF1A+ cells. Thus, pancreas agenesis enhancer mutants showed complete silencing of PTF1A in early pancreatic MPCs, yet maintain PTF1A expression in various neuronal lineages as well as in acinar cells.

### *PTF1A*^enhP^ regulation of *PTF1A* in human pancreatic MPCs

**Figure 3:**
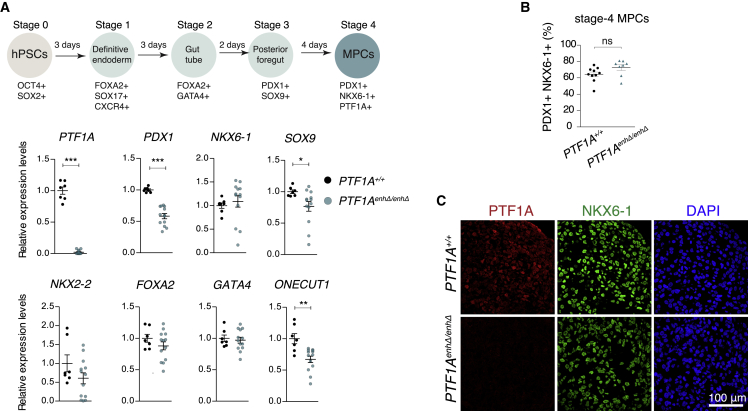
Modeling *PTF1A* enhancer mutations in human MPCs (A) qRT-PCR of human MPCs for pancreatic progenitor markers (n = 7–13 independent differentiation experiments per genotype, using 6 *PTF1A*^*enhΔ/enhΔ*^ clones—3 lines with 127 bp and 3 lines with 321-bp deletions, see Figure S2B—and 4 *PTF1A*^*+/+*^ control lines. Graphs show means ± SEM. Mann-Whitney ^∗^p ≤ 0.05, ^∗∗^p ≤ 0.01, ^∗∗∗^p ≤ 0.001). (B) Quantification of FACS data for PDX1+ NKX6-1+ stage-4 *in vitro* derived MPCs (n = 8–10 independent differentiation experiments per genotype; ns, not significant). (C) Immunofluorescence of human MPCs (stage 4) shows absence of PTF1A in *PTF1A*^*enhΔ/enhΔ*^ lines, without changes in NKX6-1. See also Figure S2.

To examine if *PTF1A*^enhP^ is also required for PTF1A expression in human pancreatic MPCs, we used H1 human pluripotent stem cells (hPSCs) to derive pancreatic MPCs (Balboa et al., 2018; Rezania et al., 2014). Pancreatic MPCs were cultured as 3D aggregates in suspension because we found that *PTF1A* and its target *CPA1* were almost undetectable with conventional adherent culture differentiation protocols, whereas *PTF1A* mRNA increased 6.8-fold in 3D aggregates (Figure S2A). We generated 6 clonal hPSC lines containing homozygous *PTF1A*^enhP^ either 127 or 322-bp deletions and differentiated them for 12 days to pancreatic MPCs, along with 3 control clonal hPSC lines generated using non-targeting gRNAs and non-modified parental H1 cells (Figures S2B–S2E). All *PTF1A*^enhP^ deleted cells formed MPCs expressing characteristic markers (*PDX1*, *NKX6-1*, *NKX2*-*2*, *ONECUT1*, *FOXA2*, and *GATA4*), with moderately reduced *PDX1* mRNA (Figures 3A–3C, S2F, S2G). *PTF1A* mRNA and protein, however, were severely reduced (Figures 3A and 3C). This indicates that in analogy to mouse studies, the distal enhancer sequence mutated in humans with pancreas dysgenesis is not required for the specification of human pancreatic MPCs but is essential to activate *PTF1A* in these cells.

### PTF1A regulates an evolutionary conserved program in MPCs

**Figure 4:**
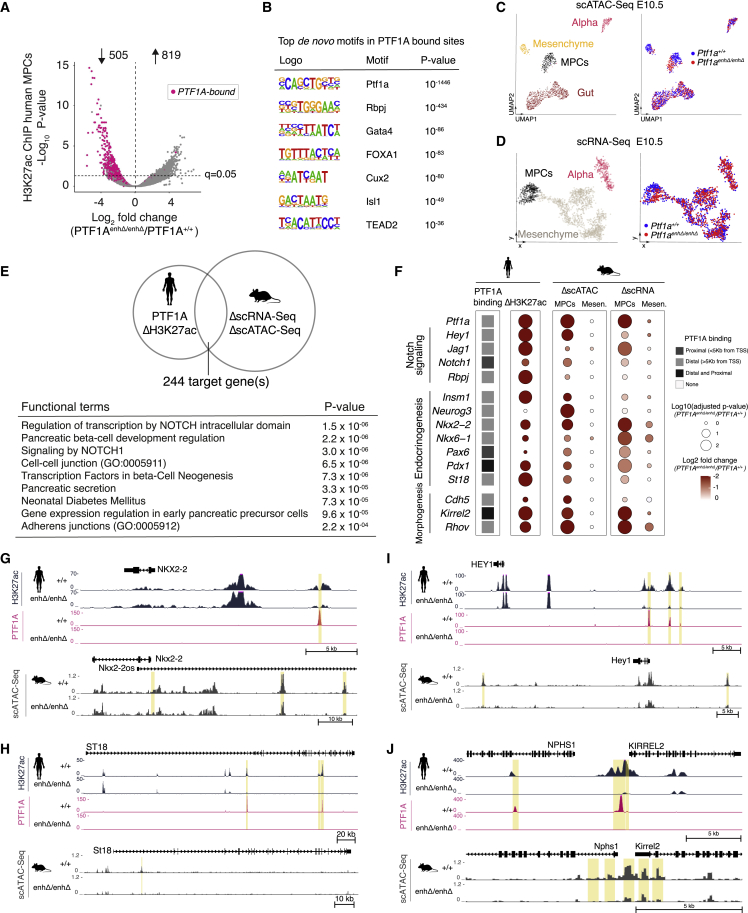
*PTF1A* regulates an evolutionary conserved program in MPCs (A) Differential analysis of H3K27ac at active regulatory regions in human *PTF1A*^*enhΔ/enhΔ*^ MPCs. Regions bound by PTF1A in MPCs are highlighted in pink. (B) Top HOMER *de novo* motifs of regions bound by PTF1A in MPCs. (C and D) (C) Uniform Manifold Approximation and Projection (UMAP) plots of scATAC and (D) metacell 2D projection of scRNA-seq from E10.5 *Ptf1a*^*enhΔ/enhΔ*^ and *Ptf1a*^*+/+*^ pancreatic buds. Both identified cells compatible with MPCs and glucagon-expressing cells (Alpha). Cells are colored by cell type (left) or genotype (right). (E) Functional enrichment of 244 genes showing PTF1A-binding or loss of H3K27ac in human mutant cells and differential accessibility or mRNA expression in mouse mutants. (F) Selected PTF1A-regulated genes in human and mouse MPCs. Mesenchymal cells (*mesen*) are shown as controls. Dot sizes represent adjusted p values, and color shade fold-change in mutant samples. (G–J) Examples of loci showing altered chromatin at PTF1A-bound regions in human mutant cells, and altered chromatin in orthologous or syntenic regions in mouse mutant E10.5 MPCs. Shown are genes involved in endocrinogenesis (*NKX2-2*, *ST18*), Notch signaling (*HEY1*), and cell adhesion (*KIRREL2-NPHS1*). Mouse tracks show aggregated MPC single-cell chromatin accessibility. See also Figures S3 and S4 and Tables S1, S2, S3, S4, and S5.

The availability of mouse and human *PTF1A*^enhP^ mutant MPCs offered a unique opportunity to characterize the genomic program that is regulated by PTF1A in MPCs. Using ChIP-seq for H3K27ac in human wild-type pancreatic MPCs, we defined 26,424 putative distal enhancers and 13,353 promoter-proximal active regulatory elements that were consistent between 2 biological replicates (Figure S3A; Table S1). This enhancer set encompassed >90% of enhancers previously defined in pancreatic MPCs from a different differentiation protocol (Cebola et al., 2015) (Figure S3B). Next, we examined these regions in *PTF1A*^*enhΔ/enhΔ*^ cells and identified *PTF1A*^enhP^-dependent H3K27ac changes, with 505 genomic regions showing decreased H3K27ac at q = 0.05, of which 94% were distal enhancers (Figures 4A and S3C–S3F; Table S2). Thus, *PTF1A*^enhP^-dependent expression of PTF1A controls an enhancer program in pancreatic MPCs.

Next, we examined which *PTF1A*^enhP^-dependent changes reflect a direct PTF1A function. Previous studies examined PTF1A binding in a mouse acinar tumor cell line (Thompson et al., 2012) or in mouse neural tube and later stages of pancreas organogenesis in which PTF1A is expressed in acinar cells (Meredith et al., 2013). Because TF binding patterns are highly cell type and stage specific, we performed ChIP-seq for PTF1A in human pancreatic MPCs. This revealed 6,768 bound regions, which were expectedly enriched for PTF1A and other pancreatic transcription factor motifs (GATA, FOXA, ONECUT, TEAD, and PDX1/ISL1) (Figure 4B; Table S1). PTF1A-bound regions showed widespread decreased H3K27ac in *PTF1A*^enhP^ mutant cells, and >85% of regions that showed a reduction in H3K27ac were bound by PTF1A (Figures 4A and S3F). Thus, 430 genomic regions were directly bound by PTF1A and showed decreased H3K27ac in human *PTF1A*^*enhΔ/enhΔ*^ MPCs. These represent direct functional PTF1A targets in human MPCs (Table S2).

To focus on important PTF1A effector sites, we searched for evolutionary conserved PTF1A-dependent changes in mouse *PTF1A*^enhP^ mutant MPCs. We thus performed single cell Assay for Transposase Accessible Chromatin sequencing (scATAC-seq) an single cell RNA sequencing (scRNA-seq) in *Ptf1a*^*enhΔ/enhΔ*^ and control E10.5 dissected buds. After defining clusters of MPCs, we identified 3,461 regions with decreased chromatin accessibility (q < 0.1, and fold-change < −0.5) and 461 downregulated mRNAs (q < 0.05) in mutant cells (Figures 4C, 4D, and S3G–S3N; Table S3). We then focused on genes that showed direct PTF1A-dependent chromatin changes in human MPCs, as well as PTF1A-dependent changes in chromatin activity or mRNA in orthologous or syntenic sites in mouse embryos. This revealed 244 loci with evolutionary conserved PTF1A-dependent changes (Figure 4E; Table S4).

The conserved PTF1A-dependent effector program was strongly enriched in genes important for β cell development and signaling (Figures 4E and 4F; Table S5). β cell development genes were not necessarily expected, as MPCs have not yet acquired an endocrine phenotype. However, PTF1A bound at numerous genes encoding transcription factors that are essential for pancreatic endocrine differentiation, including some that are not fully active in MPCs. PTF1A binding was thus detected at *INSM1*, *ST18*, *PAX6*, *NEUROG3*, *NKX2-2*, *NKX6-1*, *PDX1*, and *MNX1*, most of which showed concomitant conserved chromatin changes in MPCs (see examples for *INSM1*, *PAX6*, *PDX1*, *ST18*, and *NKX2-2*) (Figures 4G, 4H, and S4A–S4E). Thus, PTF1A directly controls an endocrine transcription factor program in pancreatic MPCs.

Remarkably, PTF1A also bound at an extensive list of canonical Notch signaling genes in human MPCs (*NOTCH1*, *RBPJ*, *JAG1*, *JAG2*, *DLL1*, *DLL4*, *MIB1*, *HEY1*, *HES1*, and *HEDLG1*), several of which showed decreased RNA and/or chromatin accessibility in mouse single-cell profiles (Figures 4I and S4F–S4H). Therefore, one of the key functions of PTF1A in MPCs may be to activate a Notch signaling program in the pancreatic epithelium. Given the profuse binding of PTF1A to endocrine regulators, it is noteworthy that Notch signaling inhibits endocrinogenesis in the first transition but subsequently promotes progenitor proliferation and endocrine cell formation (Ahnfelt-Rønne et al., 2012; Jensen et al., 2000; Shih et al., 2012).

Finally, PTF1A showed direct binding to genes encoding regulators of cell adhesion, migration, and morphogenesis including *KIRREL2*, *ITGB1*, *SLIT3*, *RHOV*, *SEMA3C*, *CTNNA2*, and *CTNND1*, encoding p120 catenin (Figures 4J, S4I, and S4J), and also targeted several T2D susceptibility loci that showed reduced activity in mouse and human knockout models (*STARD10*, *TCF7L2*, and *CDKAL1*; Table S4).

These studies, therefore, uncovered an evolutionary conserved genetic program that is controlled by *PTF1A*^enhP^ in pancreatic multipotent progenitors and revealed a major role of PTF1A to remodel chromatin at genes important for pancreas endocrinogenesis.

### *Ptf1a* enhancer deletion causes defective pancreas growth and tubulogenesis

During normal mouse development, PTF1A expression is rapidly extinguished around E11.5 as MPCs transition to bipotent duct-endocrine progenitors, which are located in the so-called “trunk” of the embryonic pancreas, whereas PTF1A expression is selectively activated in more peripheral pro-acinar “tip” cells (Jørgensen et al., 2007). The trunk progenitors form a tubular plexus that is subsequently remodeled to form the mature ductal tree (Larsen and Grapin-Botton, 2017; Pan and Wright, 2011). We examined how PTF1A function in early MPCs influenced these morphogenetic processes.

At E10.5–E11.5, the size of *Ptf1a*^*enhΔ/enhΔ*^ pancreatic buds was moderately reduced (Figures 2C and S5A; Video S1), but subsequent growth was severely stunted, with decreased phospho-Histone H3+ progenitors at E11.5 and E12.5, consistent with defective proliferation (Figures S5B–S5D). By E12.5, mutant embryos displayed clearly abnormal tubulogenesis, with a reduced number of microlumens (Figure S5E). Phosphorylated myosin light chain, which regulates contractility associated with tubulogenesis and tight junctions, was undetectable in mutants, whereas apical expression of Muc1 and E-cadherin staining were conserved (Figures S5E and S5F). From E12.5 onward, lumens became progressively more dilated, and there was reduced branching of peripheral ducts (Figure S5E). Thus, *PTF1A*^enhP^ is dispensable for the specification of pancreatic progenitors but is required for subsequent tubulogenesis, expansion of the plexus, and normal ductal branching—consistent with known morphogenetic functions of many PTF1A targets. These morphological abnormalities plausibly underpin defective pancreatic growth in patients with *PTF1A*^enhP^ mutations.

### PTF1A in MPCs primes bipotent and endocrine progenitor differentiation

**Figure 5:**
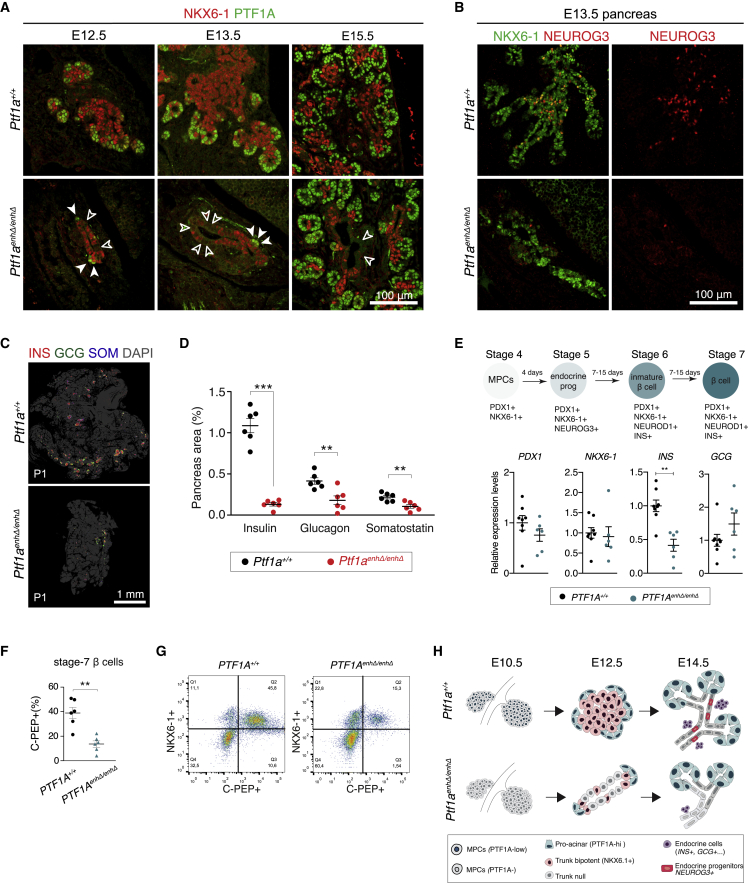
PTF1A in MPCs primes endocrine differentiation of mouse bipotent trunk progenitors (A) E12.5-15.5 pancreas showing NKX6-1 (red) in “trunk” bipotent duct-endocrine progenitors, and PTF1A (green) in peripheral pro-acinar cells. White empty arrows point to NKX6-1 negative poorly differentiated trunk cells in *Ptf1a*^*enhΔ/enhΔ*^ pancreas. White solid arrowheads depict PTF1A-positive tip cells in *Ptf1a*^*enhΔ/enhΔ*^ pancreas. (B) NEUROG3+ endocrine progenitors (red) are severely reduced in E13.5 *Ptf1a*^*enhΔ/enhΔ*^ pancreas (see also Figure S5J). (C and D) Insulin (INS), glucagon (GCG), and somatostatin (SOM) immunofluorescence of neonatal (P1) and E18.5 pancreas showed reduced endocrine cells. A representative section from P1 is shown in (C), whereas (D) shows quantifications of the relative pancreas area occupied by each endocrine cell type in E18.5 (n = 6/genotype; ^∗∗^p ≤ 0.01, ^∗∗∗^Welch’s t-test p ≤ 0.0001). (E) qRT-PCR of endocrine markers in human hPSC-derived beta-like cells (n = 6–8 independent differentiations/genotype, using 6 *PTF1A*^*enhΔ/enhΔ*^ and 4 *PTF1A*^*+/+*^ control lines). Error bars represent mean ± SEM. Mann-Whitney test, ^∗∗^p < 0.01. (F and G) (F) Flow cytometry for C-peptide expressing beta-like cells in differentiated control and mutant S7 stem cell islets (Mann-Whitney test, ^∗∗^p < 0.01), and (G) representative FACS plots (n = 6 independent differentiations/genotype). (H) Schematic summarizing the differentiation phenotype in *Ptf1a*^*enhΔ/enhΔ*^ pancreas. See also Figure S5.

Next, we examined how PTF1A function in MPCs influences differentiation of pancreatic lineages. In mouse embryos, pancreatic endocrine cells are formed in two distinct waves. The so-called “first transition” (E9.5–E11.5), which does not make a major contribution to definitive endocrine cells, was not disrupted by PTF1A deficiency in MPCs. The mass of glucagon+ cells at E10.5 was thus preserved and even mildly increased when compared with the smaller size of the mutant buds (Figures 5G and 5H).

In the “secondary transition” (E13.5–E16.5), “trunk” bipotent progenitors turn into endocrine-committed NEUROG3+ progenitors, which then differentiate to endocrine cells (Gradwohl et al., 2000; Larsen and Grapin-Botton, 2017; Pan and Wright, 2011; Pictet et al., 1972). The trunk bipotent progenitors normally express HNF1B, SOX9, NKX6-1, and NKX2.2 (Hald et al., 2008; Seymour et al., 2007; Solar et al., 2009), and no longer express PTF1A. In *Ptf1a*^*enhΔ/enhΔ*^ E12.5–E13.5 embryos, HNF1B/SOX9-expressing trunk cells lined the lumen of dilated tubular structures, but a significant fraction of these cells (hereafter, trunk *null* cells) failed to express NKX6-1 (Figure 5A) or NKX2.2 and often lacked nuclear HES1 (Figure S5I), a mediator of canonical Notch signaling. Thus, bipotent duct-endocrine progenitors showed abnormal differentiation. Because trunk *null* cells retained expression of ductal markers HNF1B and SOX9, they could represent differentiated duct cells. In consequence, the formation of endocrine NEUROG3+ progenitors from this epithelium was drastically reduced in E12.5 and E13.5 *Ptf1a*^*enhΔ/enhΔ*^ embryos (Figures 5B and S5J). This, in turn, led to severely reduced all endocrine cell types at birth (α cells 0.21% ± 0.04% versus 0.41% ± 0.04%, β cells 0.13% ± 0.02% versus 1.09% ± 0.09% of pancreas area in *Ptf1a*^*enhΔ/enhΔ*^ versus control littermates, Welch’s t test, p < 0.001) (Figures 5C, 5D, and S5K).

To assess if PTF1A also regulates human endocrine differentiation, we differentiated human *PTF1A*^*enhΔ/enhΔ*^ and control hPSCs clones to islet endocrine cells (6 mutant lines, 4 control lines, n = 6–8 independent differentiations/genotype). In line with the mouse phenotype, we observed decreased expression of *INS* mRNA, reduced numbers of C-peptide+ cells (38.9% ± 5.6% versus 13.6% ± 3.2%, p = 0.004, Mann-Whitney), as well as decreased insulin content and glucose-stimulated insulin release in *PTF1A*^*enhΔ/enhΔ*^ stem cell-derived islets (Figures 5E–5G and S5L–S5O).

In marked contrast to the endocrine differentiation defect, mutant mouse embryos showed strong PTF1A activation in tip cells after E11.5 (Figures 5A and S1H). By E13.5, these PTF1A+ cells formed proto-acini that expanded normally and drained into small ducts that showed normal expression of NKX6-1 (Figure 5A). Thus, stage-specific PTF1A deficiency in MPCs did not block subsequent pancreas development or PTF1A activation in the acinar lineage, yet caused a severe perturbation of the differentiation of bipotent duct-endocrine progenitors. This in turn caused a major decrease in the formation of endocrine cells in mouse and human *PTF1A*^*enhP*^-deficient models (Figure 5H).

### PTF1A in MPCs regulates a primed chromatin state in trunk cells

**Figure 6:**
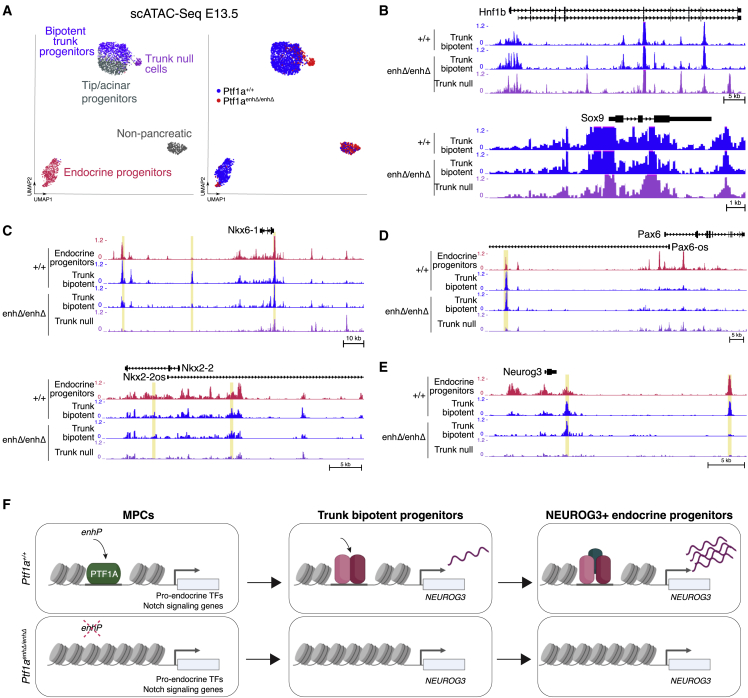
PTF1A in MPCs triggers sequential chromatin changes in *Neurog3* (A) scATAC UMAP plots of E13.5 *Ptf1a*^*enhΔ/enhΔ*^ and *Ptf1a*^*+/+*^ pancreas identifies NEUROG3+ endocrine progenitors, pro-acinar progenitors, trunk bipotent progenitors, and mutant-specific trunk *null* cells. Nuclei are colored by cell type (left) or genotype (right). (B–E) Pseudo-bulk scATAC-seq profiles from E13.5 *Ptf1a*^*+/+*^ and *Ptf1a*^*enhΔ/enhΔ*^ trunk and trunk *null* cells. Regions downregulated in trunk *null* cells (log_2_-fold-change < −0.5, binomial test FDR < 0.1) are highlighted in yellow. (B) Depicts *Hnf1b* and *Sox9* loci and (C–E) show reduced accessibility in *Ptf1a*^*enhΔ/enhΔ*^ trunk *null* cells at indicated sites of endocrine regulatory loci. Profiles in NEUROG3+ cells are shown for comparison. In (E), E13.5 *Ptf1a*^*+/+*^ trunk progenitors exhibit an active chromatin state at *Neurog3* that is similar to NEUROG3+ progenitors, whereas this is abrogated in *Ptf1a*^*enhΔ/enhΔ*^ trunk *null* cells and is altered at several sites in other *Ptf1a*^*enhΔ/enhΔ*^ trunk cells. (F) Proposed model illustrating sequential steps triggered by *PTF1A*^enhP^ activation of *PTF1A*. PTF1A binds and remodels chromatin at pro-endocrine gene loci in MPCs. Active chromatin states are maintained at endocrine genes such as *NEUROG3* in bipotent progenitor trunk cells, enabling full activation of *NEUROG3* in endocrine-committed progenitors. *PTF1A*^enhP^ deletion prevents this process, causing reduced endocrine differentiation. See also Figure S6.

To understand how transient PTF1A expression in MPCs regulates bipotent progenitors, we performed scATAC-seq in control and *Ptf1a*^*enhΔ/enhΔ*^ mouse E13.5 pancreas (Figure 6A). In both genotypes, we identified clusters of trunk bipotent duct-endocrine progenitors, NEUROG3+ pro-endocrine progenitors, and pro-acinar cells (Figure 6A). We further identified a *Ptf1a*^*enhΔ/enhΔ*^-specific cluster that was consistent with bipotent duct-endocrine progenitors, with active chromatin at typical trunk marker genes such as *Hnf1b or Sox9* (Figure 6B) but lacked active chromatin at *Nkx6-1* and *Nkx2-2* (Figures 6C and S6; Table S6). This profile was consistent with abnormally differentiated *Ptf1a*^*enhΔ/enhΔ*^ trunk *null* cells observed in immunolocalization studies (Figure 5A).

Given that PTF1A has a direct impact on the chromatin states of endocrine regulatory genes in early MPCs, we focused on essential endocrine regulatory loci in the subsequent “trunk” stage. In addition to the abnormalities described above at *Nkx6-1* and *Nkx2-2* loci (Figure 6C), *Ptf1a*^*enhΔ/enhΔ*^ trunk-like cells showed abnormal inactive chromatin sites at *Pax6*, an endocrine regulator that is not yet expressed in trunk progenitors (Figure 6D). Furthermore, we observed that wild-type trunk bipotent progenitors exhibited an active chromatin profile at the *Neurog3* locus that resembled that of NEUROG3^+^ endocrine-committed progenitors, thus showing that trunk progenitors are epigenetically primed for endocrine differentiation (Figure 6E). In *Ptf1a*^*enhΔ/enhΔ*^ embryos, however, all trunk-like cells showed abnormal accessibility at distal *Neurog3* sites, which was more pronounced in trunk *null* cells (Figure 6E). Thus, bipotent progenitors exhibit a primed enhancer landscape at key endocrine gene loci. Taken together, these findings point to a model whereby the activation of *PTF1A*^*enhP*^ sets in motion an epigenetic cascade in which transient PTF1A expression in MPCs creates a permissive chromatin state for pro-endocrine genes in bipotent progenitors, which in turn enables full activation of NEUROG3 in endocrine-committed progenitors and subsequent formation of endocrine cells (Figure 6E).

### *PTF1A*^enhP^ activates an enhancer cluster in MPCs

Loss of function of single enhancers generally fails to cause severe phenotypes due to redundancy among enhancers that regulate the same gene (Cannavò et al., 2016; Hay et al., 2016; Osterwalder et al., 2018; Spielmann et al., 2018). To understand how a single enhancer defect could have a severe phenotypic impact, we investigated the regulatory landscape of *PTF1A* in control and *PTF1A*^enhP^-defective cells.

**Figure 7:**
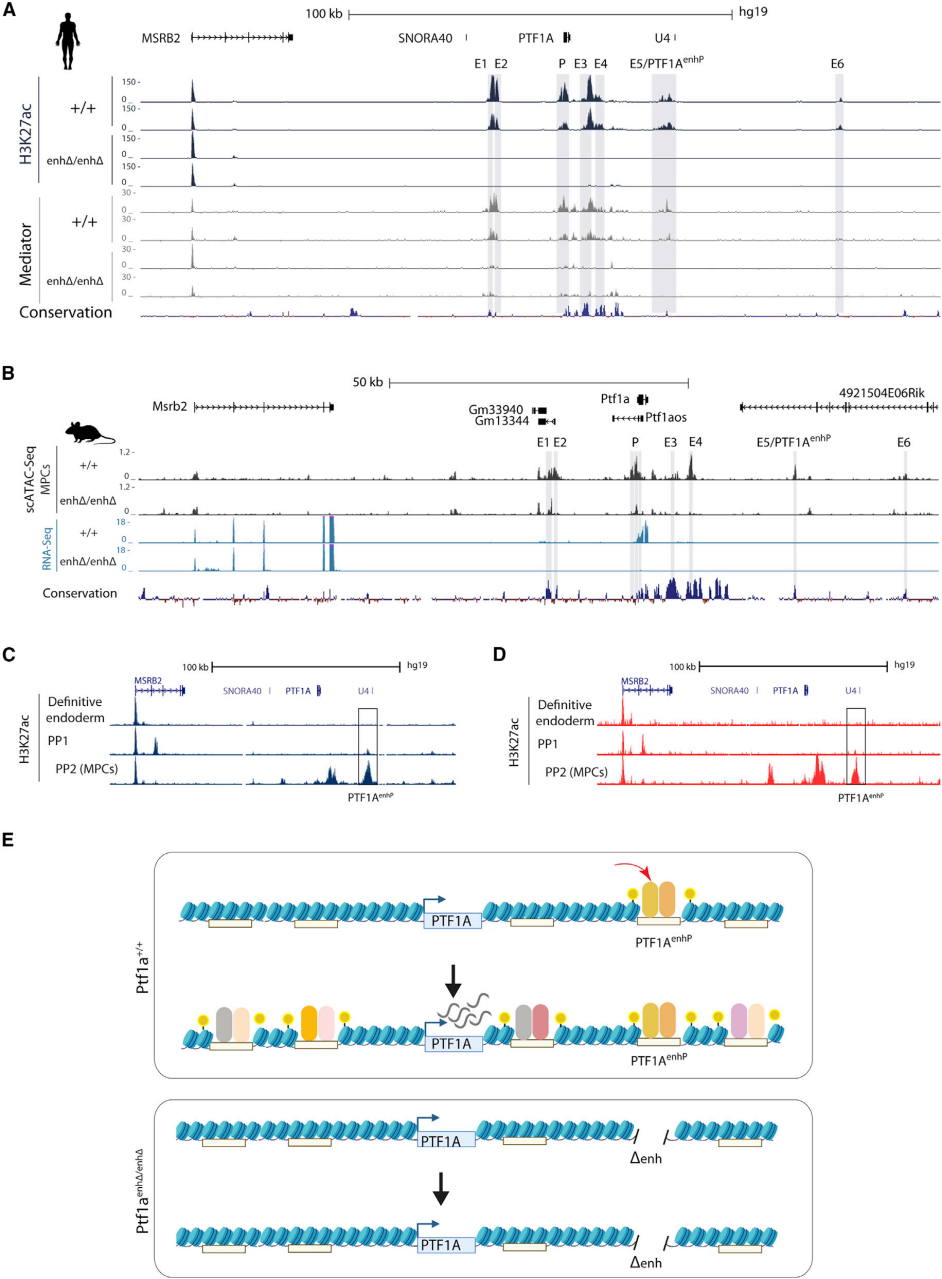
*PTF1A*^enhP^ creates an active enhancer cluster in mouse and human MPCs (A) Regulatory landscape of the human *PTF1A* locus in *PTF1A*^*+/+*^ and *PTF1A*^*enhΔ/enhΔ*^ MPCs. Six H3K27ac-enriched putative enhancers and the *PTF1A* promoter, most of which show strong mediator (MED1) binding, are shaded in gray. All show absent activity in *PTF1A*^*enhΔ/enhΔ*^ MPCs (q < 0.05). ChIP-seq tracks show a MACS2 −log_10_ p values. (B) scATAC-seq profiles for MPCs from *Ptf1a*^*+/+*^ and *Ptf1a*^*enhΔ/enhΔ*^ E10.5 pancreatic buds showed chromatin accessibility at *Ptf1a* and E1-E6 regions orthologous to human enhancers, highlighted in gray. All showed loss in *Ptf1a*^*enhΔ/enhΔ*^ cells (q < 0.1, log_2_FC < −0.5). Conservation tracks show multiple alignments between 100 vertebrate species. (C and D) H3K27ac at the *PTF1A* locus in 2 hPSC-derived pancreatic progenitor datasets (Alvarez-Dominguez et al., 2020; Geusz et al., 2021). Both used a protocol that generates two stages of early pancreatic progenitors: PP1 PDX1+ cells that do not express MPC markers such as NKX6-1, PP2 PDX1+, and NKX6.1+ cells that are comparable with stage 4 MPCs from the current study. In both datasets, H3K27ac enrichment at *PTF1A*^enhP^ preceded that of all other enhancers. (E) Summary model illustrating how *PTF1A*^enhP^ precedes and activates the enhancer cluster in the *PTF1A* locus. See also Figure S7.

Earlier studies of in hPSC-derived pancreatic MPCs showed that *PTF1A*^enhP^ is bound by PDX1 and FOXA2 and flanked by H3K4me1 (Weedon et al., 2014; Cebola et al., 2015), although bulk CS16-18 fetal pancreas did not show active enhancer modifications (Gerrard et al., 2020) (Figure S7A). We profiled H3K27ac and Mediator binding in human MPCs and identified six putative active enhancers (E1–E6) at the *PTF1A* locus, one of which corresponds to *PTF1A*^enhP^ (Figure 7A). The underlying sequences were evolutionary conserved, and all showed chromatin accessibility peaks in single-cell ATAC-seq from E10.5 mouse pancreatic MPCs (Figure 7B). Two of these enhancer regions have been previously characterized in acinar or neural lineages (Masui et al., 2008; Meredith et al., 2009; Mona et al., 2016, 2020). Consistent with findings in human bulk fetal pancreas, ATAC-seq in bulk pancreatic buds showed barely detectable accessibility at these sites, plausibly because MPCs are a small fraction of cells in dissected buds (Figure S7B). These studies, therefore, revealed the landscape of active enhancers at *PTF1A* in human and mouse pancreatic MPCs.

We also examined the specificity of active chromatin at *PTF1A*^enhP^. In keeping with the highly selective mouse mutant phenotype, *PTF1A*^enhP^ lacked active chromatin in mouse embryonic neural tube or in postnatal and E17.5 pancreas (Figures S7C and S7D). Likewise, active enhancer modifications were absent in adult human pancreas and across a broad range of adult and fetal tissues (Figures S7E–S7G) (Domcke et al., 2020; Zhang et al., 2021). All of these cell types showed signs of enhancer activity in other regions, including the E1–E2 region previously shown to direct transcription in acinar and central nervous system (Masui et al., 2008) (Figures S7C–S7F). The *PTF1A*^enhP^ enhancer, therefore, is extremely specific for mouse and human pancreatic MPCs.

Because mouse and human *PTF1A*^enhP^ mutations were not compensated by other enhancers, we examined the chromatin activity of other enhancers in the *PTF1A* cluster in mutant MPCs. It is important to note that mouse and human mutant MPCs are bona fide progenitors, albeit PTF1A deficient, as they express multiple canonical MPC markers (see for example Figures 3A and S2A) and are capable of sustaining pancreas development. In MPCs derived from the *PTF1A*^enhP^ deletion clonal stem cell lines, all six enhancers (E1–E6) and the *PTF1A* promoter lacked H3K27ac marks or Mediator binding, whereas the adjacent *MSRB2* gene remained undisturbed (Figure 7A). Similarly, scATAC profiles in MPCs from E10.5 *Ptf1a*^*enhΔ/enhΔ*^ pancreas showed that chromatin accessibility at all E1–E6 enhancers was abolished (Figure 7B). Thus, *PTF1A*^enhP^ is essential to activate of the entire PTF1A enhancer cluster in mouse and human pancreatic MPCs.

We next assessed the temporal sequence of activation of enhancers in the *PTF1A* cluster. We analyzed two chromatin datasets from hPSC-derived early PDX1+ pancreatic precursors and PDX1+, NKX6.1+ pancreatic MPCs (Alvarez-Dominguez et al., 2020; Geusz et al., 2021). In both datasets, the earliest PDX1+ precursors lacked H3K27ac enrichment at all *PTF1A* domain enhancers except the *PTF1A*^enhP^ enhancer, which showed weak H3K27 acetylation (Figures 7C and 7D). Taken together, these results indicate that *PTF1A*^enhP^ is selectively switched on early pancreatic MPCs, and this step is essential to activate the *PTF1A* enhancer cluster (Figure 7E). Therefore, failure to activate *PTF1A*^enhP^ prevents the formation of the enhancer cluster, precluding functional redundancy by shadow enhancers.

## Discussion

Our understanding of how non-coding variants can influence human disease has major gaps. The current study demonstrates that a non-coding element that is mutated in patients with pancreas agenesis is a *cis*-acting enhancer whose only role is to activate a developmental enhancer cluster. This regulatory event in turn sets in motion an epigenetic cascade that is required for normal differentiation of pancreatic endocrine cells. This analysis reveals functional hierarchy in the formation of an enhancer cluster and identifies a mechanism whereby loss-of-function enhancer mutations circumvent functional redundancy.

PTF1A is transiently expressed in early progenitors of the embryonic pancreas, but its molecular function at this stage is poorly understood. The selective nature of the *PTF1A*^*enhP*^ mutant phenotype, which did not completely disrupt pancreas organogenesis, allowed us to dissect the PTF1A-dependent genomic program in MPCs. Our findings indicate that *PTF1A*^enhP^ initiates a multi-step mechanism that drives pancreatic duct and endocrine differentiation (Figure 6E). First, transcription of *PTF1A* in MPCs leads to direct PTF1A binding and chromatin activation at numerous loci encoding regulators of endocrine differentiation. This in turn primes an endocrine-competent chromatin state in subsequent bipotent duct-endocrine progenitors. Notably, bipotent progenitors exhibit active chromatin at the *Neurog3* locus, in anticipation for full activation of the *Neurog3* gene in endocrine-committed progenitors. Importantly, this entire process was dependent on prior exposure to PTF1A in MPCs. Thus, the *PTF1A* enhancer initiates a sequence of epigenetic priming events in MPCs and their progeny that is required for normal endocrine differentiation (Figure 6E).

This function contrasts with the established essential role of PTF1A in the differentiation of exocrine acinar cells (Krapp et al., 1996, 1998). Interestingly, *PTF1A*^*enhP*^ is inactive in pancreatic acinar cells (Figures S7D and S7E) and was not required to activate *PTF1A* in this lineage, indicating that unlike MPCs, pro-acinar cells contain transcription factors required to activate other enhancers at the *PTF1A* locus.

These findings have overarching ramifications for the differentiation of hPSCs into functional β cells. Despite recent progress, *in vitro* protocols do not yet fully recapitulate progenitor populations and endocrine differentiation pathways (Bakhti et al., 2019). *In vitro* protocols have been typically benchmarked with a limited set of marker genes, including PDX1 and NKX6-1, without due consideration of other key pancreatic MPC regulators such as PTF1A (Nair et al., 2020; Wesolowska-Andersen et al., 2020). Furthermore, differentiation protocols have largely neglected the bipotent “trunk” duct-endocrine differentiation stage, a key step in the formation of bona fide endocrine cells. Our work highlights the importance of PTF1A in early MPCs to enable subsequent formation of endocrine-competent progenitors and provides new gene regulatory milestones to improve efforts to generate β cells *in vitro*.

This study also has implications for understanding the functional heterogeneity of transcriptional enhancers. Epigenomics has revealed hundreds of thousands of human enhancer-like regions, but it is unlikely that they are functionally equivalent. Our findings reveal an example of an enhancer that acts as a master key to activate a cluster of active enhancers and is therefore functionally distinct. Interestingly, this distinction was based on functional criteria, and unlike the definition of super-enhancers (Whyte et al., 2013), it did not exhibit unique epigenomic features that stand out from other enhancers in the cluster. It is intriguing to speculate that *PTF1A*^enhP^ could activate other enhancers by promoting the recruitment of the locus to subcellular condensates or other transcription factor-rich microenvironments (Grosveld et al., 2021; Hnisz et al., 2017).

Finally, these findings have relevance for human genetics. Several studies have raised questions concerning the scope of enhancer mutations in Mendelian disease, given that deletion of individual enhancers often does not elicit severe consequences due to the redundant nature of multi-enhancer domains (Cannavò et al., 2016; Hay et al., 2016; Kvon et al., 2021; Osterwalder et al., 2018; Spielmann et al., 2018). The *PTF1A* progenitor enhancer sets a paradigm for an unknown fraction of enhancers in the human genome that are vulnerable to loss-of-function Mendelian mutations because of a hierarchical role in the activation of enhancer domains at critical biological stages.

### Limitations of the study

The current study uncovered an individual enhancer that activates all other enhancers in the same regulatory cluster; however, further studies are needed to define the signals that trigger activation of the lead enhancer in the first place. The study also does not shed light on how many enhancers in the human genome have a similar leading function. Finally, the main conclusions of the current study are based on convergent findings from mouse *in vivo* models and human *in vitro* models. It is reasonable to conclude that the findings should translate to human pancreas development, although this has not been directly tested *in vivo*.

## STAR★Methods

### Key resources table

**Table:** 

REAGENT or RESOURCE	SOURCE	IDENTIFIER
**Antibodies**		

Rabbit polyclonal H3K27ac - ChIP	Abcam	Cat# ab4729; RRID:AB_2118291
Rabbit polyclonal MED1 - ChIP	Bethyl Laboratories	Cat# A300-793A; RRID:AB_577241
Goat Anti-Mouse polyclonal Carboxypeptidase a1 - IF	R and D Systems	Cat# AF2765, RRID:AB_2085841
Rabbit anti-mouse monoclonal Cleaved Caspase-3 (Asp175) (5A1E) - IF	Cell Signaling Technology	Cat# 9664, RRID:AB_2070042
Rabbit anti-mouse monoclonal CDX2 (D11D10) - IF	Cell Signaling Technology	Cat# 12306, RRID:AB_2797879
Mouse monoclonal Anti-Cadherin, E - IF	BD Biosciences	Cat# 610181, RRID:AB_397580
Guinea Pig polyclonal Anti-Glucagon - IF	Millipore	Cat# 4031-01F, RRID:AB_433707
Polyclonal Rabbit Anti-Human Glucagon - IF	Dako (now Agilent)	Cat# A0565, RRID:AB_10013726
Rabbit monoclonal HES1 (D6P2U) - IF	Cell Signaling Technology	Cat# 11988, RRID:AB_2728766
Rabbit polyclonal HNF-1beta (H-85), discontinued- IF	Santa Cruz Biotechnology	Cat# sc-22840, RRID:AB_2279595
Goat anti-human polyclonal HNF1B/TCF2 - IF	Novus	Cat# NB100-57093, RRID:AB_843965
Guinea pig polyclonal Insulin - IF	Dako (now Agilent)	Cat# A0564, RRID:AB_10013624
Goat anti-mouse polyclonal Kirrel2/NEPH3 - IF	R and D Systems	Cat# AF2930, RRID:AB_2265276
Mouse monoclonal MNR2/HB9/Mnx1 - IF	DSHB	Cat# 81.5C10, RRID:AB_2145209
Rabbit polyclonal Mucin 1 (H-295), discontinued - IF	Santa Cruz Biotechnology	Cat# sc-15333, RRID:AB_2148554
Sheep Anti-Human polyclonal Neurogenin-3 - IF	R and D Systems	Cat# AF3444, RRID:AB_2149527
Mouse monoclonal Nkx2.2 - IF	DSHB	Cat# 74.5A5, RRID:AB_531794
Mouse monoclonal Nkx6.1 - IF	DSHB	Cat# F55A10, RRID:AB_532378
Mouse monoclonal Pax6 - IF	DSHB	Cat# pax6, RRID:AB_528427
Rabbit monoclonal non-phospho (Active) β-Catenin (Ser45) (D2U8Y) – IF	Cell Signaling Technology	Cat# 19807, RRID:AB_2650576
Mouse Anti-Rat monoclonal Pdx1 IF	DSHB	Cat# F6A11, RRID:AB_1157904
Rabbit polyclonal Phospho-Histone H3 (Ser10) - IF	Cell Signaling Technology	Cat# 9701, RRID:AB_331535
Rabbit polyclonal Phospho-Myosin Light Chain 2 (Ser19) - IF	Cell Signaling Technology	Cat# 3671, RRID:AB_330248
Goat polyclonal Somatostatin (D-20), discontinued - IF	Santa Cruz Biotechnology	Cat# sc-7819, RRID:AB_2302603
Polyclonal Sox9 – IF	Millipore	Cat# AB5535, RRID:AB_2239761
Rabbit Glu2 -IF	Gift from Bernard Thorens' lab (UNIL)	NA
Guinea pig Hb9/Mnx1 -IF	Gift - Samuel Pfaff’s lab (Salk Institute)	NA
Guinea pig Neurogenin3 – IF	Gift - Michael S German's lab (UCSF)	NA
Mouse Pdx1 – IF	Gift – Chris Wright’s lab (Vanderbilt University)	NA
Goat Ptf1a – IF	Gift – Chris Wright’s lab (Vanderbilt University)	NA
Rabbit Ptf1a – IF	Gift - Bertrand Blondeau’s lab (UPMC)	NA
Mouse Anti-CD184 (CXCR4) Monoclonal Antibody, Phycoerythrin Conjugated, Clone 12G5 - Flow cytometry	BD Biosciences	Cat# 555974; RRID:AB_396267
Mouse IgG2a, kappa Isotype Control, Phycoerythrin Conjugated, Clone G155-178 antibody - Flow cytometry	BD Biosciences	Cat# 563023; RRID:AB_2716792
Mouse Anti-PDX1 Phycoerythrin Conjugated - Flow cytometry	BD Biosciences	Cat# 562161; RRID:AB_10893589
Mouse Anti-NKX6-1 Alexa Fluor 647 Conjugated - Flow cytometry	BD Biosciences	Cat# 563338; RRID:AB_2738144
Mouse Anti-NKX6.1 Phycoerythrin Conjugated - Flow cytometry	BD Biosciences	Cat# 555574; RRID:AB_395953
Mouse-Anti-C-peptide-647 - Flow cytometry	BD Biosciences	Cat# 565831; RRID:AB_2739371
Mouse IgG1, kappa Isotype Control, Phycoerythrin Conjugated, Clone MOPC-21 - Flow cytometry	BD Biosciences	Cat# 555749; RRID:AB_396091
Mouse IgG1 kappa isotype control Alexa 647 Conjugated - Flow cytometry	BD Biosciences	BD Biosciences Cat# 557714; RRID:AB_396823

**Chemicals, peptides, and recombinant proteins**		

Ascorbid acid	Sigma	Cat# A4544
Nicotinamide	Sigma	Cat# N0636
Heparin	Sigma	Cat# H3149-25KU
Zinc Sulfate	Sigma	Cat# Z0251-100G
NaHCO3	Sigma	Cat# S5761
Glucose	SIGMA	Cat# G8769-100ML
BSA	Lampire	Cat# 7500804
Insulin-Transferrin-Selenium-Ethanolamine (ITS -X) (100X)	Life Technologies	Cat# 51500-056
Sodium pyruvate solution 100mM	Merck lifescience SLU	Cat# S8636-100ML
Chemically Defined Lipid Concentrate	Life Technologies	Cat# 11905031
Trace Elements A	Corning	Cat# 25-021-CI
Trace Elements B	Corning	Cat# 25-022-CI
GlutaMax	Life Technologies	Cat# 35050038
MCDB 131 Medium, no glutamine	Life Technologies	Cat# 10372-019
Corning™ CMRL 1066	Fisher Scientific	Cat# Corning™ 15-110-CV
Y-27632 2HCl (ROCKi)	Selleckchem	Cat# S1049
Matrigel Growth Factor Reduced	Corning	Cat# 356231
0.5 mM EDTA	ThermoFisher	Cat# 15575
FGF7	Genscript	Cat# Z03407-1
CHIR	Tocris	Cat# 4423
ActivinA	Qkine	Cat# QK001 ActA_1000
Human EGF	Peprotech	Cat# AF-100-15
Retinoic acid	Sigma	Cat# R2625
TPB (amyloid precursor protein modulator)	Santa Cruz Biotechnology	Cat# sc-204424
SANT-1	Sigma	Cat# S4572
LDN-193189	Selleckchem	Cat# S2618
RepSox	Selleckchem	Cat# S7223
Human Betacellulin	Peprotech	Cat# 100-50-100UG
T3	Sigma	Cat# T6397-100MG
N-Acetyl Cysteine (NAC)	Sigma	Cat# A9165-5G
ZM447439	Selleckchem	Cat# S1103
Gamma Secretase Inhibitor XX (GSiXX)	Calbiochem	Cat# 565789
Exendin-4	Enzo	Cat# ENZ-PRT111-0001
Essential 8	Life Technologies	Cat# A1517001

**Critical commercial assays**		

Glucomen Areo glucose sensing strips	Menarini Diagnostics	Cat#47950
Ultra Mouse Insulin ELISA Kit	Crystal Chem	Cat#90080
Insulin High Range Assay kit	Perkin Elmer - cisbio	Cat#62IN1PEG
DirectPCR Lysis Reagent	Viagen Biotech	Cat#102-T
DreamTaq Green PCR Master Mix	Thermo Fisher Scientific	Cat#K1081
Phusion Green High-Fidelity DNA Polymerase	Thermo Fisher Scientific	Cat#F534S
Normal Donkey Serum	Jackson ImmunoResearch	Cat#017-000-121
TSA amplification kit	Invitrogen/Molecular Probes	Cat#T30955
Fluorescence Mounting Medium	Dako (now Agilent)	Cat#S3023
UltraPure™ Low Melting Point Agarose	Thermo Fisher Scientific	Cat# 16520050
benzyl alcohol	Sigma	Cat#108006-100ML
benzyl benzoate	Sigma	Cat#B6630-250ML
Antibody Diluent with Background Reducing Components	Dako (now Agilent)	Cat#S3022
NucleoSpin RNA Plus Kit	Macherey Nagel	Cat# 22740984.50
Transcriptor First Strand cDNA Synthesis Kit	Life Science Roche	Cat#04897030001
LightCycler 480 SYBR Green IMasterMix	Life Science Roche	Cat# 04707516001
SMART-Seq v4 Ultra low input RNA kit	Takara Bio	Cat#634890
Chromium Single Cell 3′ Library & Gel Bead Kit v3	10x Genomics	Cat#PN-1000075
Chromium Single Cell ATAC Library & Gel Bead Kit	10x Genomics	Cat#PN-1000110

**Deposited data**		

Human MPCs ChIP-seq raw and processed reads	This paper	GEO: GSE183674
Human MPCs ChIP-seq processed reads	This paper	Mendeley Repository, https://doi.org/10.17632/xnckzpgxj8.1
Mouse MPCs scRNA-Seq and scATAC-Seq	This paper	GEO: GSE183674

**Experimental models: Cell lines**		

Human: H1 (WA01)human embryonic stem cell line (hPSCReg ID: WAe001-A; NIH approval number NIHhESC-10-0043)	WiCell	Cat# WA01

**Experimental models: Organisms/strains**		

Mouse: C57BL/6J	The Jackson Laboratory	RRID: IMSR_JAX:000664
Mouse: Tg(Sox9-EGFP)EB209Gsat/Mmucd	MMRRC (Gong et al., 2003)	RRID:MMRRC_011019-UCD
Mouse: Ptf1a n.567_837del line (abbreviated to Ptf1aenhΔ/enhΔ)	This paper	NA

**Oligonucleotides**		

RNA sequence: mouse Ptf1a enhancer 5’gRNA1: ATCAGCCACACAATGTAATC	This paper	NA
RNA sequence: mouse Ptf1a enhancer 3’gRNA1: CCCTTCAATTGGCCGTTTTT	This paper	NA
RNA sequence: human PTF1A enhancer 5’gRNA1: GACTCTTTAAGTGGCTTGTC	This paper	NA
RNA sequence: human PTF1A enhancer 5’gRNA2: TGTCAATAGCACCTAATTAC	This paper	NA
RNA sequence: human PTF1A enhancer 3’gRNA3: GTAATTATCCAAGAGAGCAC	This paper	NA
RNA sequence: human PTF1A enhancer 3’gRNA4: TTAATCCCCTTAGAGTAACA	This paper	NA
qRT-PCR PTF1A F/R (TTATCCGAACAGCCAAAGTCTGGACC; AGTCTGGGACCTCTCAGGACACAA)	This paper	NA
qRT-PCR PDX1 F/R (AAGTCTACCAAAGCTCACGCG; CGTAGGCGCCGCCTGC)	This paper	NA
qRT-PCR NKX6-1 F/R (TATTCGTTGGGGATGACAGAG; TGGCCATCTCGGCAGCGTG)	This paper	NA
qRT-PCR SOX9 F/R (ATCAAGACGGAGCAGCTGAG; GGCTGTAGTGTGGGAGGTTG)	This paper	NA
qRT-PCR NKX2-2 F/R (GAACCCCTTCTACGACAGCA; ACCGTGCAGGGAGTACTGAA)	This paper	NA
qRT-PCR FOXA2 F/R (AAGACCTACAGGCGCAGCT; CATCTTGTTGGGGCTCTGC)	This paper	NA
qRT-PCR GATA4 F/R (GAGGAAGGAGCCAGCCTAGCAG; CGGGTCCCCCACTCGTCA)	This paper	NA
qRT-PCR ONECUT1 F/R (CGCTCCGCTTAGCAGCATG; GCTGGACATCTGTGAAGACC)	This paper	NA
qRT-PCR KI67 F/R (CGACCCTACAGAGTGCTCAA; TGCTCCTTCACTGGGGTCTT)	This paper	NA
qRT-PCR INS F/R (CAGAAGCG TGGCATTGTGGA; GCTGCGTCTAGTTGCAGTAG)	This paper	NA
qRT-PCR GCG F/R (GAAGGCGAGATTTCCCAGAAG; CCTGGCGGCAAGATTATCAAG)	This paper	NA
qRT-PCR NEUROG3 F/R (GACGACGCGAAGCTCACCAA; TACAAGCTGTGGTCCGCTAT)	This paper	NA

**Software and algorithms**		

HOMER	Heinz et al., 2010	http://homer.salk.edu/homer/
Cutadapt v1.9.1	Martin, 2011	https://pypi.python.org/pypi/cutadapt/1.9.1
Trimgalore v0.4.1	NA	https://www.bioinformatics.babraham.ac.uk/projects/trim_galore/
Bowtie2 v2.1.0	Langmead and Salzberg, 2012	http://bowtie-bio.sourceforge.net/bowtie2/index.shtml
Samtools v1.2	Li et al., 2009	http://samtools.sourceforge.net
BEDTools	Quinlan and Hall, 2010	https://github.com/arq5x/bedtools2
MACS2	Zhang et al., 2008	https://github.com/taoliu/MACS
bedgraphToBigWig	NA	https://github.com/ENCODE-DCC/kentUtils
DESeq2 v1.10.1	Love et al., 2014	https://www.bioconductor.org/packages/DESeq2
Cellranger v3.0.2	10x Genomics	https://support.10xgenomics.com/single-cell-gene-expression/software/overview/welcome
Cellranger-atac v1.2.0	10x Genomics	https://support.10xgenomics.com/single-cell-atac/software/overview/welcome
Seurat v3.1.4	Stuart et al., 2019	https://github.com/satijalab/seurat
Signac v1.1.0	Stuart et al., 2021	https://github.com/timoast/signac/
Sinto v0.4.2	NA	https://timoast.github.io/sinto/
ImageJ	Schneider et al., 2012	https://imagej.nih.gov/ij/

**Other**		

Infinite M200 plate reader	Tecan	NA
MZ16F stereomicroscope	Leica	NA
SP5 confocal microscope	Leica	NA
Single Plane Illumination Microscopy	Luxendo MuVi SPIM CS	NA
NEPA21 Super Electroporator	Nepagene	NA
LightCycler® 480 Instrument II	Roche	NA
Bioanalyzer	Agilent	NA
Islet perifusion chamber	BIOREP Technologies	Cat# PERI-CHAMBER
Perifusion chamber filter	BIOREP Technologies	Cat# PERI-FILTER
Perifusion chamber rubber ring	BIOREP Technologies	Cat# PERI-O-RING
Perifusion steel nozzle	BIOREP Technologies	Cat# PERI-NOZZLE
8-channel peristaltic pump	ISMATEC	Cat# ISM931A
Two-stop color code tube 0.38mm ID Tygon R3607	ISMATEC	Cat# 070534-03i / SC0003
Connecting tube 1.016mm ID Tygon 3603	ISMATEC	Cat# SC0035

### Resource availability

#### Lead contact

Further information and requests for resources and reagents should be directed to and will be fulfilled by the lead contact, Jorge Ferrer (jorge.ferrer@crg.eu).

#### Materials availability

All unique/stable reagents generated in this study are available from the lead contact with a completed materials transfer agreement.

### Experimental model and subject details

#### Ethics statement

Animal experimentation was carried out in compliance with EU Directive 86/609/EEC and Recommendation 2007/526/EC regarding the protection of animals used for experimental and other scientific purposes, enacted under Spanish law 1201/2005, and also approved by the institutional ethics committee. Human embryonic stem cells experiments were authorized by the Health Department, Generalitat de Catalunya (register number 0336/2443/2019) after ethical and methodological approval by the Center of Regenerative Medicine in Barcelona and the Institute of Health Carlos III (Ministry of Science and Innovation) in compliance with European and National regulations. European Research Council Advanced Grant (789055) funds were not used for human ESC experiments.

#### Generation of CRISPR-Cas9 mice carrying a *Ptf1a* enhancer deletion

The orthologous mouse sequence of the human pancreas agenesis *PTF1A* enhancer (*PTF1A*^*enhP*^) was deleted *in vivo* using CRISPR-Cas9 editing in the hybrid B6CBAF1 mouse strain (Charles River). Pairs of CRISPR guide RNAs (gRNAs) were designed using an online tool (http://crispr.mit.edu/) to minimize off-targets and to target PAM sequences located upstream and downstream of the enhancer sequence. Forty ng/μl gRNAs (5’gRNA1 ATCAGCCACACAATGTAATC and 3’gRNA1 CCCTTCAATTGGCCGTTTTT) and 75 ng/ μl Cas9mRNA were injected in B6CBAF1 embryos at pronuclear stage by the Transgenics Facility from MRC London Institute of Medical Sciences-Imperial College London, generating several founders. F0 founder mice were genotyped using primers spanning the deleted regions, which were validated and mapped using Sanger sequencing (Figures S1A–S1C). F1 Ptf1a n.567_837del 393nt mice carrying the deletion (abbreviated to *Ptf1a*^*enhΔ*^) were backcrossed with C57BL/6J (Charles River) mice for 7 generations and heterozygous mice were intercrossed to generate *Ptf1a*^*enhΔ/enhΔ*^ or *Ptf1a*^*+/+*^ littermates.

Tail tips from adult mice were lysed using a Proteinase K-containing lysis buffer. Embryo biopsies were lysed using DirectPCR Lysis Reagent (Viagen Biotech). DreamTaq Green DNA Polymerase (Thermo Scientific) and the following primers, which span the deleted enhancer region, were used for genotyping: Ptf1a-outside1-F (5’-TCTCCAAAAGCTTTCAAGTGACC-3’) and Ptf1a-outside1-R (5’-TTATACCAGAGATGGCCTGCC-3’), The cycling programme used was 95^°^C for 5 min, followed by 35 cycles of 95^°^C 30 sec, 58^°^C 30C and 72^°^C for 1 min, with final extension step of 10 min at 72^°^C, which generates a 290bp product in case of a deletion and a 610 bp band in WT alleles (see Figure S1B).

#### hPSC lines and differentiation protocol

Human embryonic stem cell line H1 (WA01, Wicell) was cultured on Matrigel-coated (Corning) plates in Essential 8 (E8) medium (Thermo Fisher) and passaged using EDTA. Differentiation to pancreatic progenitors and endocrine cells was conducted following a previously published protocol (Balboa et al., 2022). To start the differentiation experiments, stem cells were dissociated to single cells with EDTA and seeded at 0.22 million cells/cm^2^ on Matrigel coated plates in E8 medium containing 5 μM ROCK inhibitor (Selleckchem). Differentiation was started the day after using a seven-stage differentiation protocol: stages 1 to 3 (definitive endoderm, primitive gut tube and posterior foregut) were induced in adherent culture; then cells were dissociated and reaggregated in suspension culture in rotation and cultured during stage 4 (multipotent pancreatic progenitors), stage 5 (endocrine progenitors), stage 6 (endocrine cells) and stage 7 (endocrine cell maturation).

#### Genome editing of PTF1A enhancer deletions in hPSCs

*PTF1A* enhancer region was deleted in H1 hESC using CRISPR-SpCas9 ribonucleoproteins (Alt-R S.p. HiFi Cas9 Nuclease V3, 77522245, Integrated DNA Technologies (IDT). Two pairs of guide RNAs flanking the enhancer region were designed using Benchling (Biology Software, 2019) and Custom Alt-R® CRISPR-Cas9 guide RNA (IDT), synthesized as crRNA and complexed with tracrRNA (Alt-R® CRISPR-Cas9 tracrRNA, ATTO™ 550, 1075928, IDT).

Guide RNA pair 1+4 (gRNA1: GACTCTTTAAGTGGCTTGTC; gRNA4: TTAATCCCCTTAGAGTAACA) generates a 321 bp deletion and guide RNA pair 2+3 (gRNA2: TGTCAATAGCACCTAATTAC; gRNA3: GTAATTATCCAAGAGAGCAC) generates a 127 bp deletion (Figures S2B and S2C). Ribonucleoprotein complexes were prepared according to manufacturer's instructions and delivered to one million stem cells by electroporation (NEPA21 Super Electroporator, Nepagene). Electroporated cells were plated, expanded and single cell cloned by FACS-assisted single cell deposition. Stem cell clones were expanded, processed for DNA extraction with DirectPCR Lysis Reagent (Viagen Biotech) and screened for *PTF1A* enhancer deletions using PCR with Phusion High-Fidelity DNA polymerase (Thermo Fisher), primers spanning the deleted enhancer region (PTF1A-human-enhancer-F: TTAAAACAACAGGGGCAACTGAAC; PTF1A-human-enhancer-R: TATGTCCTTCCTAGGCTGGTT, amplicon size 744 bp) and a touch-down thermal cycle (98C for 3 m; 8 cycles of 98C for 10 s, 66C to 62C (decreasing 0.5C every cycle) for 30 s, 72C for 22 s; 30 cycles of 98C for 10 s, 62C for 30 s, 72C for 22 s and a final extension step of 8 m at 72C. Deleted alleles generated a 423 bp (using gRNAs 1+4) or 617 bp (using gRNAs +3) amplicon (see Figures S2D and S2E). A total of 6 clonal cell lines with homozygous enhancer deletion were generated: 3 lines with g1+g4 combinations (321 bp deletion, KO1, KO2, KO3) and 3 lines generated with g2+g3 (127 bp deletion, KO4, KO5, KO6). These are referred to as *PTF1A*^*enhΔ/enhΔ*^ in the manuscript. They were used for differentiation experiments together with 4 control cell lines: 3 clonal cell lines generated with non-targeting gRNAs NC1 CGTTAATCGCGTATAATACG and NC2 CATATTGCGCGTATAGTCGC, CTRL1, CTRL2 and CTRL3) and the parental cell line H1, all together referred to as *PTF1A*^*+/+*^.

### Method details

#### Glycemic measurements and meal tests

Glycemia measurements were taken by tail tip sampling using the Glucomen Areo glucometer and glucose sensing strips (Menarini Diagnostics). For meal tests, hyperglycaemic *Ptf1a*^*enhΔ/enhΔ*^ and *Ptf1a*^*+/+*^ 7 week-old male littermates were used (n=7-8/genotype). Animals were fasted overnight and blood glucose levels were measured after fasting and 1 hour after *ad libitum* feeding. Circulating insulin was quantified from 5μl of plasma in technical duplicates using the Ultra Mouse Insulin ELISA Kit (Crystal Chem), following manufacturer’s instructions. Measurements were acquired by the Infinite M200 plate reader (Tecan) and standard curves were fitted using a quadratic polynomial regression.

#### *In toto* immunohistochemistry of mouse embryos

Embryos were fixed in 4% paraformaldehyde overnight at 4^°^C washed in PBS and stored in 100% methanol (MeOH) at -20C. They were then were incubated in Dent’s bleach (MeOH:DMSO:H2O2, 4:1:1) overnight at room temperature to bleach pigmented cells and to reduce autofluorescence. Then, embryos were washed in pure methanol, brought to -80^o^C and thawn to RT between 3 and 5 times for at least 1h each time to render antigens in the deeper parts of the tissues accessible. After repeated freezing and thawing, samples were hydrated stepwise in PBST (PBS + 0.2% Triton) and washed thoroughly in this medium. Embryos were blocked overnight at room temperature in 1X blocking buffer (a proprietary TSA-block supplied by Invitrogen) containing 10% heat inactivated serum from the same species in which the secondary antibody was derived (i.e. for Donkey anti-rabbit secondary antibodies, donkey serum, Jackson ImmunoResearch Laboratories, Inc. was used). This buffer was used for all antibody incubation steps. After blocking, the embryos were incubated in primary antibody for 72h at 4^°^C (see key resources table) and washed extensively for 24h. All washing steps were performed with gently rocking and at least 5 washes with PBST buffer. Incubation with secondary antibody was performed as described for the primary antibody but with additional filtering (0.45μm) to remove potential artifacts from fluorophore precipitates. For the detection of PTF1A, TSA amplification kit (Perkin-Elmer) was used following manufacturers’ recommendations except for the incubation time with the substrate, which was extended up to 20 minutes to facilitate penetration into the sample. Following TSA or secondary antibody incubations and washes, the specimens were washed in distilled water, mounted in low melting agarose (Invitrogen), transferred stepwise to MeOH and cleared in BABB (benzyl alcohol, (Sigma) and benzyl benzoate (Sigma) in a 1:2 ratio) for at least 24h. Images were acquired using a Single Plane Illumination Microscopy (Luxendo MuVi SPIM CS). 3D reconstruction from serial pictures obtained with SPIM was performed with the 3D Project tool of the ImageJ software (Schneider et al., 2012).

#### Immunofluorescence

Embryos, adult tissues and cells were processed for immunofluorescence analysis after paraffin embedding as previously described (Maestro et al., 2003). Briefly, tissues were fixed in 4% paraformaldehyde overnight at 4^°^C while rotating, washed in PBS and embedded in paraffin. Paraffin blocks were sectioned in 4 μm sections, deparaffinized with xylene and rehydrated through an ethanol series. Sections were incubated for 30 min at room temperature in antibody diluent (DAKO Corporation) with 3% normal serum from the same species as the secondary antibody, and incubated overnight at 4C with the primary antibody of interest (see key resources table). The following day sections were followed incubated overnight at 4^°^C with the secondary antibody, stained with DAPI and mounted with Dako Fluorescence Mounting Medium (Molecular Probes, S3023). Images were acquired using a Leica SPE confocal microscope.

#### Endocrine mass quantification

For endocrine cell mass measurements, 6 *Ptf1a*^*enhΔ/enhΔ*^ and 6 *Ptf1a*^*+/+*^ perinatal pancreases were dissected and embedded in paraffin. Three paraffin sections of 4 μm width were obtained from each pancreas at 300 μm intervals throughout the organ. Immunofluorescence was performed for insulin, glucagon, somatostatin and DAPI as described above (for antibodies see key resources table). Images were automatically captured and 10x10 frames were reconstructed using an SP5 confocal microscope (Leica). The area for each hormone was quantified by establishing the same value in the "threshold" ImageJ plug-in for *Ptf1a*^*enhΔ/enhΔ*^ and *Ptf1a*^*+/+*^ pancreas samples and they were expressed as the percentage of the total area of the pancreas obtained by saturating the DAPI signal.

#### Quantification of proliferation

To establish differences in proliferation between *Ptf1a*^*enhΔ/enhΔ*^ and *Ptf1a*^*+/+*^ MPCs immunofluorescence was performed with antibodies directed against the pancreatic transcription factors PDX1, NKX6.1 and the proliferation marker pHH3, essentially as described above. We used 4 *Ptf1a*^*+/+*^ and 4 *Ptf1a*^*enhΔ/enhΔ*^ E11.5 pancreas, for which we analyzed at least 3 sections taken at 20 μm intervals. Results were expressed as the percentage of PDX1, NKX6.1+ cells that were pHH3-positive.

#### Single-cell RNA-Seq and ATAC Seq libraries

Dissected E10.5 and E13.5 embryonic pancreases were dissociated to single cells using Accutase solution (Sigma) and immediately used to build scRNA-Seq libraries (Chromium Single Cell 3′ Library Gel Bead Kit v3, 10x Genomics) or to extract nuclei for scATAC-Seq libraries (Chromium Single Cell ATAC Library Gel Bead Kit, 10X Genomics), following manufacturers' instructions with a target recovery of 5000 cells/nuclei per sample. A pool of 9 *Ptf1a*^*+/+*^ or *Ptf1a* ^*enhΔ/enhΔ*^ embryos was used to generate E10.5 scRNA-Seq datasets; 8 *Ptf1a*^*+/+*^ or 5 *Ptf1a* ^*enhΔ/enhΔ*^ embryos for E10.5 scATAC-Seq datasets and 6 *Ptf1a*^*+/+*^ or *Ptf1a* ^*enhΔ/enhΔ*^ embryos to create E13.5 scATAC-Seq datasets.

#### scRNA-Seq analysis

Sequencing reads were aligned to the mm10 reference genome and quantified using *cellranger count* (10x Genomics, v3.0.2). A total of 6,724 *Ptf1a*^*+/+*^ and 8,435 *Ptf1a* ^*enhΔ/enhΔ*^ cells from E10.5 pancreatic buds were sequenced. Cells with less than 300 genes were considered not informative and were removed. To minimize doublets, we filtered cells with more than 30,000 genes per cell and to minimize potentially dead cells, we filtered cells with more than 12.5% of mitochondrial counts.

For downstream analyses, we excluded hemoglobins and mitochondrial genes from the data. We retained 5,055 *Ptf1a*^*+/+*^ and 6,261 *Ptf1a*^*enhΔ/enhΔ*^ single cells, with a median UMI count of 9,519 and 9,485; and median gene counts of 1,767 and 2,048 for *Ptf1a*^*+/+*^ and *Ptf1a*^*enhΔ/enhΔ*^, respectively (Figures S3K and S3L). We used Metacell package (Baran et al., 2019) to select feature genes, construct cell clusters (termed metacells), and visualize single cell RNA-seq data. We selected feature genes using normalized size correlation threshold of -0.1 and normalized niche score threshold of 0.05, additionally filtering for genes with >2 UMI in at least three cells and a total gene UMI count >100 molecules. We excluded from the feature gene lists imprinted genes and other genes highly correlated to them. For kNN graph building we used K=100 target number of edges per cell, and for metacell construction we used K=30, minimum module size of 10, and 500 iterations of bootstrapping with resampling 75% of the cells. This way we obtained an estimate of co-clustering frequency between all pairs of single cells and identified robust clusters of single or grouped metacells (Figure S3M). We calculated per-metacell gene expression fold-change (FC) used in the downstream analyses as a regularized geometric mean within each metacell, divided by the median across metacells. Finally, we annotated metacells based on the expression FC of known marker genes.

Differentially accessible genes were determined using exact Fisher test with Benjamini Hochberg correction (Figure S5N; Table S3).

#### scATAC-Seq analysis

Sequencing reads were aligned to the mm10 reference genome and quantified using *cellranger-atac count* (10x Genomics, v1.2.0). A total of 4,318 *Ptf1a*^*+/+*^ and 4,232 *Ptf1a*^*enhΔ/enhΔ*^ nuclei from E10.5 pancreatic buds, and 6,859 *Ptf1a*^*+/+*^ and 2,998 *Ptf1a*^*enhΔ/enhΔ*^ nuclei from E13.5 were sequenced. Both *Ptf1a*^*+/+*^ and *Ptf1a* ^*enhΔ/enhΔ*^ datasets showed a strong nucleosomal banding pattern and enrichment of chromatin accessibility signal around TSS (see Figures S3G and S3H). Nuclei with less than 3,000 reads, less than 15% of reads in peaks or with low nucleosomal signal strength (below 5) were considered of low quality and were removed. Nuclei with more than 25,000 reads were also removed to minimize doublets. Cells with more than 0.05% of reads falling on blacklisted regions from ENCODE were considered artifacts and removed.

For downstream analysis we used ArchR (Granja et al., 2021). We removed barcodes with <1000 unique fragments and TSS enrichment <4, resulting in 4,601 *Ptf1a*^*+/+*^ and 4,466 *Ptf1a*^*enhΔ/enhΔ*^ cells in E10.5 dataset, and 7,219 *Ptf1a*^*+/+*^ and 3,235 *Ptf1a*^*enhΔ/enhΔ*^ cells in E13.5 dataset. We generated gene activity scores used in the downstream analyses by counting fragments per barcode per 500 bp bins, then summing fragment counts in bins overlapping a gene’s body, and applying an exponential decay weighting function to fragment counts in bins overlapping distal regulatory elements. We identified cell doublets using *in silico* simulation and clustering approach implemented in ArchR (k = 10 cells around pseudo-doublets, filterRatio = 1). We filtered out 211 (4.59%) *Ptf1a*^*+/+*^ and 199 (4.46%) *Ptf1a*^*enhΔ/enhΔ*^ cells in doublets in the E10.5 dataset, and 521 (7.22%) *Ptf1a*^*+/+*^ and 104 (3.21%) *Ptf1a*^*enhΔ/enhΔ*^ in the E13.5 dataset. We then performed dimensionality reduction using three iterations of latent semantic indexing (LSI), each sampling 4,000 cells. We used this reduced dimensions representation of the data for clustering (kNN graph construction followed by Louvain clustering with resolution = 0.5) and UMAP 2D projection (Figures 4C, 6A, and S3I). We annotated the resulting clusters by unconstrained integration with scRNA data, as implemented in ArchR. Clusters that could not be confidently annotated in this way were annotated by accessibility of known marker genes.

For visualization of individual genes’ scores on UMAP projection, we used MAGIC imputation (van Dijk et al., 2018) (k=5; Figures 6A and S3I).

Peak calling was performed with MACS2 (Zhang et al., 2008) for each cluster using the following parameters: --format BED --call-summits --keep-dup all --extsize 200 --shift -100. Differentially accessible peaks were determined by binomial test with Benjamini Hochberg correction (Figure S3J; Table S3). To find motifs enriched in differentially accessible peaks we used HOMER (Heinz et al., 2010) findMotifsGenome.pl with the following parameters: -size 250 -len 6,8,10,12, using as a background the non-differentially accessible peaks in the compared groups (Figure S3J).

#### Quantitative RT-PCR in hPSC models

Total RNA was isolated from differentiated stem cells using NucleoSpin RNA Plus (Macherey Nagel). A total of 1000 ng RNA was retrotranscribed to cDNA using Transcriptor First Strand cDNA Synthesis Kit (Roche). Gene expression was assessed by qRT-PCR using LightCycler 480 SYBR Green MasterMix (Roche) in 10 μl reactions in 384-well plates run in a LightCycler® 480 Instrument II (Roche). Relative quantification of gene expression was performed using delta-delta C_t_ method, with Cyclophilin G (PPIG) as endogenous normalization control. RT-reaction with no template (water control) was used as negative control. A positive control composed of mixture of RNA samples from different stages (Golden Control) was used as calibrator across qRT-PCR runs.

#### Flow cytometry

Flow cytometry analysis of definitive endoderm induction efficiency (%CXCR4+ cells), pancreatic progenitor (%PDX1+ and %NKX6-1+ cells) and beta cell formation (%NKX6-1+ and %C-PEP+ cells) was performed as described elsewhere (Lithovius et al., 2021). Antibodies used are presented in the key resources table.

#### Dynamic perifusion experiments of beta cell-like derivatives

Dynamic perifusion assays were performed using an Ismatec 8-channel peristaltic pump (Ismatec) and 2-stop 0.38mm tygon tubes (Fisher Scientific) connected to a perifusion chamber (Biorep Technologies) and to 1.016mm tygon tube (Fisher Scientific) and nozzle for consistent drop collection (Biorep Technologies). Approximately 100 islet-like aggregates were resuspended in Krebs Ringer Buffer at 37°C with different glucose concentrations and secretagogues at a fixed flow of 150ul/min and effluent collection every 5 minutes in 96 deep-well plate.

Insulin levels were measured from eluates collected every 5 minutes using Cisbio HTRF High Range Insulin Assay Kit (Cisbio) following manufacturers instructions, and read in a SPARK 10M multimode plate reader (Tecan). Total insulin content was measured by collecting islet-like stage-7 aggregates after the experiment, sonicating for 20 seconds in water and transferring an aliquot into acidic etanol (1.5% HCl in 100% ethanol).

#### ChIP of human MPCs

ChIP reactions were performed as previously described (Miguel-Escalada et al., 2019) using between 3-5 million MPCs derived from hPSCs as starting material and 1 μg H3K27ac antibody (Abcam ab4729), 3 μg Mediator (MED1 Antibody, A300-793A, Bethyl Laboratories) or PTF1A antibodies (Phan-Hug et al., 2008). ChIP-Seq libraries were prepared using the NEBNext® Ultra™ II DNA Library Prep Kit and sequenced using 50 bp SE reads using Illumina’s HiSeq 2500 platform.

#### ChIP-Seq analysis

H3K4me1, PDX1 and FOXA2 ChIP-Seq datasets from human MPCs were downloaded from ArrayExpress using accession number E-MTAB-1990. H3K27ac and PTF1A ChIP-Seq datasets from P4 mouse pancreas (Kalisz et al., 2020) and E17.5 mouse pancreas (Meredith et al., 2013) were downloaded from ArrayExpress (E-MTAB-7944 accession number) and GEO databases (GSE47459 accession number), respectively.

Raw sequencing reads obtained from public repositories or those generated *in house* were processed as follows. Adaptors were trimmed from raw sequencing reads with cutadapt v.1.9.1 (Martin, 2011) (options: -*m 20*). Trimmed reads were aligned to mm10 or hg19 using bowtie2 v.2.1.0 (Langmead and Salzberg, 2012) (options: *--no-unal*). Uniquely mapping reads (MAPQ ≥ 30) were retained using SAMtools v.1.9 (Li et al., 2009) and blacklisted regions and duplicates were removed. Mediator and PTF1A narrow peaks were called using --*bw=300 --keep-dup all* -*q 0.01*. H3K27ac peaks were called using –*bw*=*300 --keep-dup all* -*q 0.05* using MACS2 (Zhang et al., 2008). Consistent ChIP-Seq peaks between the 2 biological samples were defined by overlapping MACS2 called peaks from each replicate with bedtools *intersectBed* v 2.27.1 using the default 1 bp overlap (Quinlan and Hall, 2010). A list of 39,777 H3K27ac, 7,275 Mediator and 6,738 PTF1A consistent sites can be found on Table S1. ChIP-Seq peaks were assigned to genes using ChIPseeker R package (Yu et al., 2015) and considering proximal peaks (promoters) those that are located within 1Kb from the TSS of known genes annotated by UCSC (*TxDb.Hsapiens.UCSC.hg19.knownGene*). ChIP signal tracks were created using *macs2 bdgcmp* function on the bedGraph files generated by *macs2 callpeak* and -*m logLR* -*p 0.00001* options. Bigwig files were then generated using *bedGraphToBigWig* from UCSC tools.

In order to identify differential H3K27ac-enriched regions in *PTF1A*^*enhΔ/enhΔ*^ MPCs, a total of 95,173 narrow peaks were interrogated. The number of H3K27ac reads mapping to each peak was calculated using FeatureCounts v1.6.4. Then, paired DESeq2 (v1.10.1(Love et al., 2014)) analysis was used to assess differential signal strength. Peaks showing differential H3K27ac ChIP-seq signal at adjusted p < 0.05 (n=505 downregulated and 831 upregulated sites) were considered statistically significant and are listed on Table S2.

#### Motif analysis

*De novo* transcription factor motif enrichment analysis was performed with HOMER *findMotifsGenome.pl* function (Heinz et al., 2010), looking for 6-, 8-, 10-, and 12-bp sized motifs in fragments of 100 bp around the center of consistent PTF1A-bound regions. Consistent H3K27ac bound regions were used as background and statistical significance was determined using the default binomial distribution. Known transcription factor motif enrichment analysis on differential *PTF1A*^*enhΔ/enhΔ*^ H3K27ac peaks was performed with HOMER *findMotifsGenome.pl* function (Heinz et al., 2010), looking for 6-, 8-, 10-, and 12-bp sized motifs in fragments of 100 bp around the center of consistent H3K27ac dysregulated regions using as background the union of all H3K27ac peaks identified in MPCs.

#### Integrative analysis of mouse and human PTF1A-dependent programs

To define evolutionary conserved PTF1A targets in mouse and human MPCs, we assigned genomic regions to genes based on linear genomic distance as described above, and selected loci that showed **a)** differential accessibility in mouse scATAC-Seq (q < 0.1 and log_2_ fold change <0.5) or downregulated RNA in mouse scRNA-Seq (q < 0.05) from *Ptf1a*^*enhΔ/enhΔ*^ MPCs, and **b)** distal PTF1A binding (>1Kb) accompanied by decreased H3K27ac in human *PTF1A*^*enhΔ/enhΔ*^ MPCs (q <0.05), or PTF1A binding proximal to the transcriptional start site (<1Kb) regardless of H3K27ac changes. Ortholog genes for human datasets were identified using biomaRt interface (Durinck et al., 2009). A complete list of evolutionarily genomic regions and predicted gene targets can be found on Table S4.

### Statistical analysis

Statistical analysis was conducted as indicated in individual figure legends. Medians and standard error of the mean (S.E.M) are shown unless otherwise specified, and n values refer to the number of embryos/cell lines/animals analyzed per group. GraphPad Prism version 7.0.0 for Mac (GraphPad Software, USA) was used for unpaired two-sided Student's t test, Welch's t test, or Mann-Whitney test in Figures 1, 3, 5, S2 and S6. Statistical analysis for single cell and bulk genome data is described in corresponding sections of method details.

## Data Availability

Raw sequencing reads from ChIP-Seq, scRNA-Seq and scATAC-Seq have been deposited in the Gene Expression Omnibus (GEO) public repository at NCBI (https://www.ncbi.nlm.nih.gov/geo/) under accession number GSE183674. Processed files are deposited on Mendeley at https://doi.org/10.17632/xnckzpgxj8.1. scATAC-seq and scRNA-seq datasets can be interactively explored at https://sebe-lab.shinyapps.io/Ptf1a, where we show 2D projections and heatmaps of expression and accessibility for all genes across cell types. Additionally, it is possible to interactively inspect the expression of individual genes or groups of genes, and retrieve specifically expressed marker genes with user-defined thresholds. Any additional information required to reanalyze the data reported in this paper is available from the lead contact upon request.

## Supplemental information

Document S1. Figures S1–S7

Table S1. H3K27ac, mediator-enriched and PTF1A-bound regions in human MPCs, related to Figure 4 Consistent set of H3K27ac, mediator, and PTF1A-bound regions in human stage 4 MPCs.

Table S2. Differentially enriched H3K27ac regions in mutant MPCs, related to Figure 4 H3K27ac changes in *PTF1A*^*enhΔ/enhΔ*^ stage-4 MPCs. Significance was established at adjusted q = 0.05.

Table S3. Differential scATAC-seq and scRNA-seq analysis in mouse E10.5 MPCs, related to Figure 4 Differentially accessible regions or expressed mRNAs in *Ptf1a*^*enhΔ/enhΔ*^ E10.5 mouse MPCs. For scATAC significance was established at FDR < 0.1 and log_2_ fold change of 0.5. Differentially expressed genes in *Ptf1a*^enhΔ/enhΔ^ E10.5 mouse MPCs measured by scRNA-seq. Significance was established at adjusted p value < 0.05.

Table S4. Evolutionarily conserved program controlled by PTF1A in mouse and human MPCs, related to Figure 4

Table S5. Functional annotations of the PTF1A-dependent MPC program, related to Figure 4 Functional annotations of PTF1A program genes using EnrichR, with Fisher’s exact test p values and Benjamini-Hochberg correction. *Z* scores represent deviations from Enrichr pre-computed expected ranks. Terms are ranked by EnrichR combined score.

Table S6. Differentially accessible regions in E13.5 *Ptf1a*^*enhΔ/enhΔ*^ bipotent trunk cells versus control bipotent trunk cells and differentially accessible regions in E13.5 *Ptf1a*^*enhΔ/enhΔ*^ **bipotent trunk cells** versus control trunk *null* cells. Significance was established at FDR < 0.1 and log_2_ fold change of 0.5., related to Figure 6

Document S2. Article plus supplemental information
