# Supplementary material for: Pancreas agenesis mutations disrupt a lead enhancer controlling a developmental enhancer cluster
Source: Dev Cell. 2022 Aug 22;57(16):1922–1936.e9. doi: 10.1016/j.devcel.2022.07.014 (PMC9426562; doi:10.1016/j.devcel.2022.07.014)
Supplement: Document S1. Figures S1–S7 [file mmc1.pdf]

Developmental Cell, Volume *57*

Supplemental information

Pancreas agenesis mutations disrupt a lead enhancer controlling a developmental enhancer cluster

Irene Miguel-Escalada, Miguel Ángel Maestro, Diego Balboa, Anamaria Elek, Aina Bernal, Edgar Bernardo, Vanessa Grau, Javier García-Hurtado, Arnau Sebé-Pedrós, and Jorge Ferrer

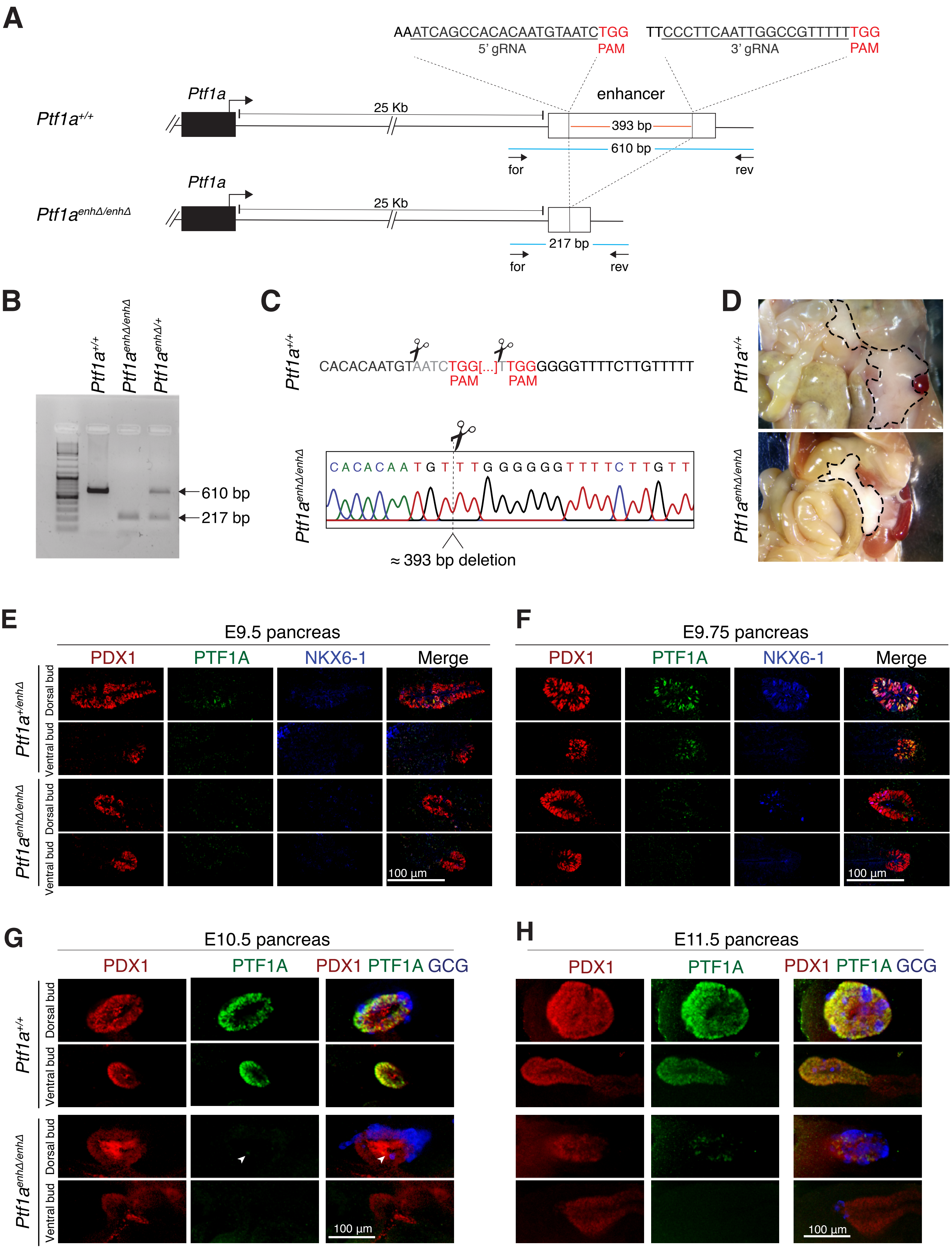

A
AAATCAGCCACACAATGTAATCTGG
5' gRNA
PAM
TTCCCTTCAATTGGCCGTTTTTTGG
3' gRNA
PAM
Ptf1a
enhancer
Ptf1a+/+
25 Kb
393 bp
610 bp
for
rev
Ptf1a
Ptf1aenhΔ/enhΔ
25 Kb
217 bp
for
rev
B
Ptf1a+/+
Ptf1aenhΔ/enhΔ
Ptf1aenhΔ/+
610 bp
217 bp
C
Ptf1a+/+
CACACAATGTAATCTGG[...]TTGGGGGGTTTTCTTGTTTTT
PAM
PAM
Ptf1aenhΔ/enhΔ
C A C A C A A T G T T T G G G G G G T T T T C T T G T T
≈ 393 bp deletion
D
Ptf1a+/+
Ptf1aenhΔ/enhΔ
E
E9.5 pancreas
PDX1
PTF1A
NKX6-1
Merge
Ptf1a+/enhΔ
Dorsal bud
Ventral bud
Ptf1aenhΔ/enhΔ
Dorsal bud
Ventral bud
100 µm
F
E9.75 pancreas
PDX1
PTF1A
NKX6-1
Merge
100 µm
G
E10.5 pancreas
PDX1
PTF1A
PDX1 PTF1A GCG
Ptf1a+/+
Dorsal bud
Ventral bud
Ptf1aenhΔ/enhΔ
Dorsal bud
Ventral bud
100 µm
H
E11.5 pancreas
PDX1
PTF1A
PDX1 PTF1A GCG
100 µm

Figure S1. CRISPR-Cas9 engineering of *Ptf1a* enhancer deletion in mouse. Related to Figures 1 and 2.
(A) Schematic of mouse *Ptf1a*^{enhP} enhancer deletion in mouse including 5'gRNA and 3'gRNA sequences and PAM sites (red) within the enhancer, leading to a 393 bp deletion. The location of genotyping primers (black arrows) as well as expected PCR product sizes (blue) are shown. **(B)** Agarose gel showing the PCR product generated when genotyping *Ptf1a* enhancer region (610 bp) in wild type, heterozygous and homozygous mice. Successful enhancer deletion generates PCR product of 217bp. **(C)** Sanger sequencing chromatogram of the region flanking *Ptf1a* enhancer deletion. PAM sequence is in red and scissors indicate breakpoints. **(D)** Brightfield image of dissected 7 week-old wild type and *Ptf1a*$^{enh\Delta/enh\Delta}$ littermates. The pancreas is delimited by a dashed line, showing hypoplasia in mutant mice. **(E-H)** Confocal images showing that PTF1A is depleted in pancreatic MPCs that form ventral and dorsal buds in E9.5 **(E),** E9.75 **(F)** and E10.5 **(G)** *Ptf1a*$^{enh\Delta/enh\Delta}$ embryos. Note that PTF1A expression in control embryos increases progressively between E9.5 and E10.5. PDX1 expression is moderately reduced in *Ptf1a*$^{enh\Delta/enh\Delta}$ pancreas. Extracellular background fluorescence in buds is indicated with an arrowhead. **(H)** At E11.5 PTF1A is detected in a few cells of *Ptf1a*$^{enh\Delta/enh\Delta}$ dorsal buds, which may represent incipient pro-acinar progenitors, while it is still absent in the ventral bud. Wild-type littermates (*Ptf1a*$^{+/+}$) or *Ptf1a*$^{+/enh\Delta}$ embryos are shown as control, as indicated.

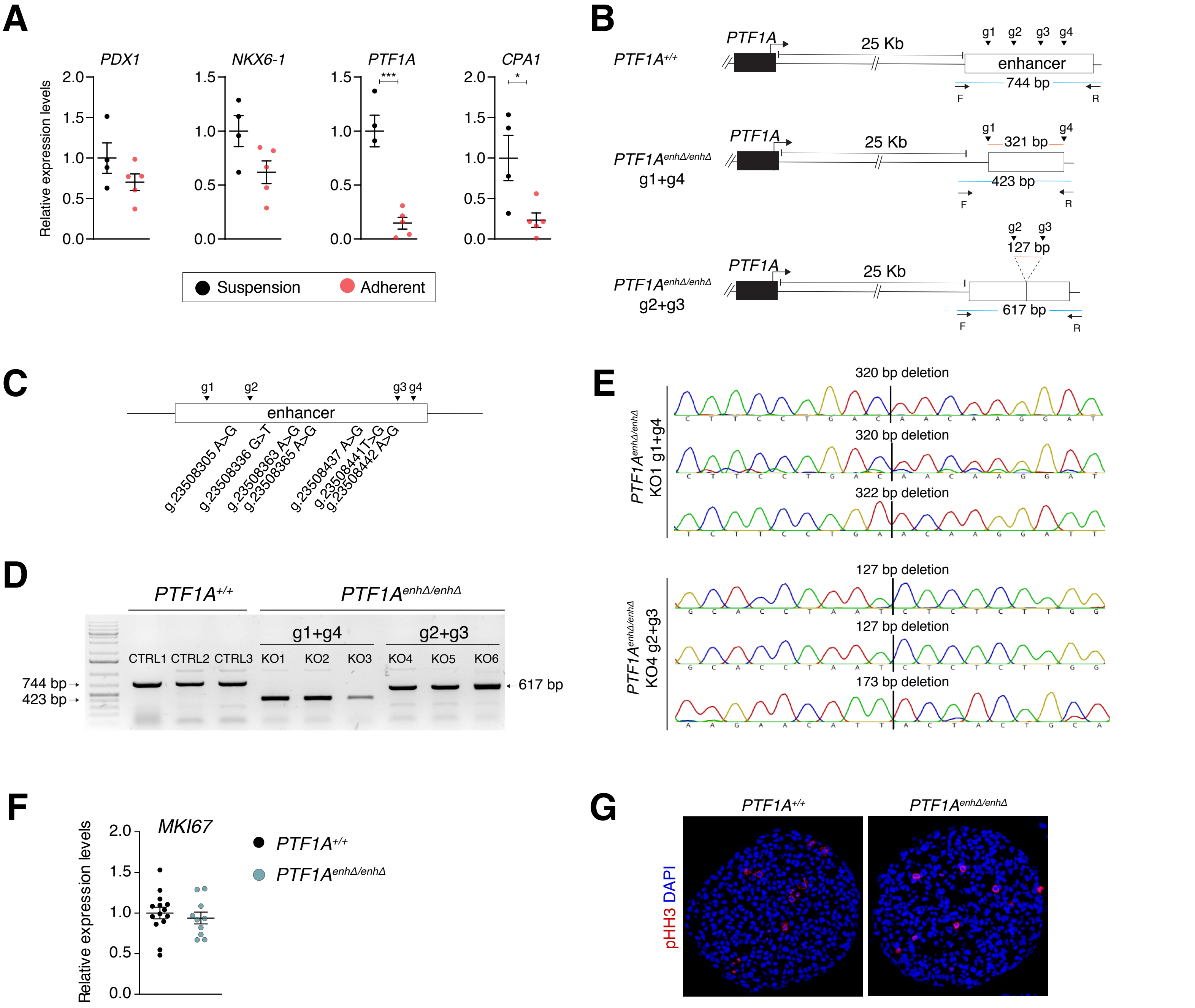

Figure S2. Modeling *PTF1A* enhancer deficiency in human pancreatic MPCs. Related to Figure 3.
(A) qRT-PCR of multipotency markers in human MPCs differentiated in suspension or adherent conditions (n = 4-5 independent differentiation experiments per condition, using 3 control hPSC lines). **(B)** Schematic of *PTF1A* enhancer deletion in hPSCs depicting gRNAs cutting sites within the enhancer (g1 to g4, black arrowheads), leading to 321 (g1+4) or 127 (g2+3) nucleotide deletions. The location of genotyping primers (arrows), as well as expected PCR product sizes (blue) are shown. **(C)** Schematic representation of the location of single base pair mutations described in human patients (Gabbay et al., 2017; Weedon et al., 2014) relative to the cut sites of gRNAs employed to generate *PTF1A*$^{enh\Delta/enh\Delta}$ lines. **(D)** Agarose gel showing the PCR product generated when genotyping *PTF1A* enhancer region (744 bp) in 3 control cell lines (*PTF1A*$^{+/+}$) and 6 homozygous cell lines with enhancer deletion (*PTF1A*$^{enh\Delta/enh\Delta}$). Successful enhancer deletion generated PCR products of 423 bp for the g1+4 deletions and 617 bp for the g2+3 deletions. **(E)** Sanger sequencing chromatogram of the region flanking *PTF1A* enhancer deletion for six *PTF1A*$^{enh\Delta/enh\Delta}$ cell lines. **(F-G)** Proliferation is not altered in *PTF1A*$^{enh\Delta/enh\Delta}$ lines. **(F)** qRT-PCR of human MPCs for MKI67 proliferation marker (n = 10 independent differentiation experiments per genotype, using 6 *PTF1A*$^{enh\Delta/enh\Delta}$ – 3 lines with 127 bp and 3 lines with 321 bp deletions. **(G)** pHH3 immunofluorescence (red) in DAPI-stained *PTF1A*$^{enh\Delta/enh\Delta}$ and control MPCs.

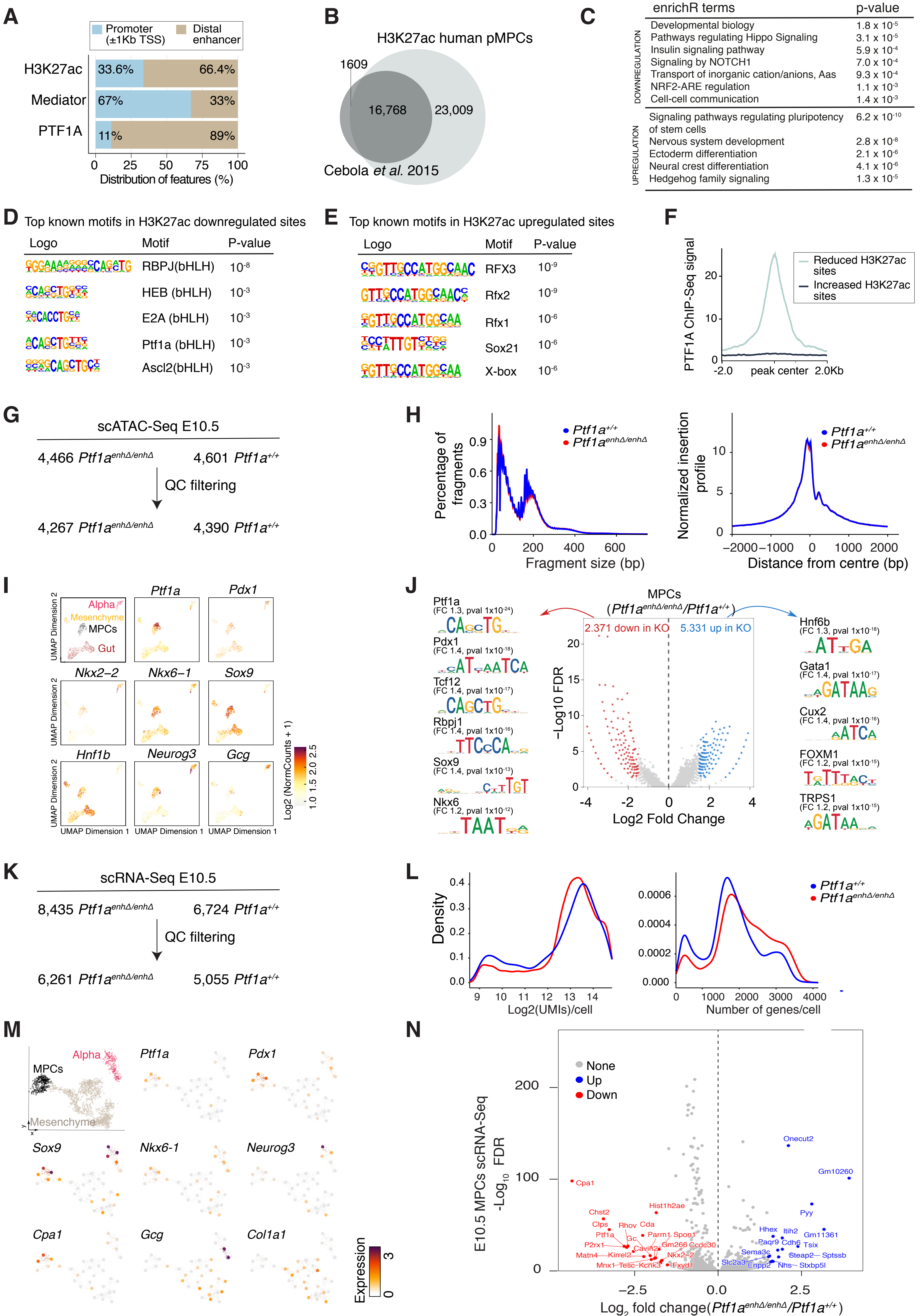
A
Promoter (±1Kb TSS)
Distal enhancer
H3K27ac
Mediator
PTF1A
33.6%
66.4%
67%
33%
11%
89%
0
25
50
75
100
Distribution of features (%)
B
H3K27ac human pMPCs
1609
16,768
23,009
Cebola et al. 2015
C
enrichR terms
p-value
DOWNREGULATION
Developmental biology 1.8 x 10-5
Pathways regulating Hippo Signaling 3.1 x 10-5
Insulin signaling pathway 5.9 x 10-4
Signaling by NOTCH1 7.0 x 10-4
Transport of inorganic cation/anions, Aas 9.3 x 10-4
NRF2-ARE regulation 1.1 x 10-3
Cell-cell communication 1.4 x 10-3
UPREGULATION
Signaling pathways regulating pluripotency of stem cells 6.2 x 10-10
Nervous system development 2.8 x 10-8
Ectoderm differentiation 2.1 x 10-6
Neural crest differentiation 4.1 x 10-6
Hedgehog family signaling 1.3 x 10-5
D
Top known motifs in H3K27ac downregulated sites
Logo
Motif
P-value
RBPJ(bHLH) 10-8
HEB (bHLH) 10-3
E2A (bHLH) 10-3
Ptf1a (bHLH) 10-3
Ascl2(bHLH) 10-3
E
Top known motifs in H3K27ac upregulated sites
Logo
Motif
P-value
RFX3 10-9
Rfx2 10-9
Rfx1 10-6
Sox21 10-6
X-box 10-6
F
PTF1A ChIP-Seq signal
Reduced H3K27ac sites
Increased H3K27ac sites
-2.0
peak center
2.0Kb
G
scATAC-Seq E10.5
4,466 Ptf1aenhΔ/enhΔ
4,601 Ptf1a+/+
QC filtering
4,267 Ptf1aenhΔ/enhΔ
4,390 Ptf1a+/+
H
Percentage of fragments
Ptf1a+/+
Ptf1aenhΔ/enhΔ
Fragment size (bp)
Normalized insertion profile
Distance from centre (bp)
I
UMAP Dimension 2
UMAP Dimension 1
Alpha
Mesenchyme
MPCs
Gut
Ptf1a
Pdx1
Nkx2−2
Nkx6−1
Sox9
Hnf1b
Neurog3
Gcg
Log2 (NormCounts + 1)
J
MPCs
(Ptf1aenhΔ/enhΔ/Ptf1a+/+)
2.371 down in KO
5.331 up in KO
-Log10 FDR
Log2 Fold Change
Ptf1a (FC 1.3, pval 1x10-24)
Pdx1 (FC 1.4, pval 1x10-18)
Tcf12 (FC 1.4, pval 1x10-17)
Rbpj1 (FC 1.4, pval 1x10-16)
Sox9 (FC 1.4, pval 1x10-13)
Nkx6 (FC 1.2, pval 1x10-12)
Hnf6b (FC 1.3, pval 1x10-18)
Gata1 (FC 1.4, pval 1x10-17)
Cux2 (FC 1.4, pval 1x10-16)
FOXM1 (FC 1.2, pval 1x10-15)
TRPS1 (FC 1.2, pval 1x10-15)
K
scRNA-Seq E10.5
8,435 Ptf1aenhΔ/enhΔ
6,724 Ptf1a+/+
QC filtering
6,261 Ptf1aenhΔ/enhΔ
5,055 Ptf1a+/+
L
Density
Log2(UMIs)/cell
Number of genes/cell
Ptf1a+/+
Ptf1aenhΔ/enhΔ
M
Alpha
MPCs
Mesenchyme
Ptf1a
Pdx1
Sox9
Nkx6-1
Neurog3
Cpa1
Gcg
Col1a1
Expression
N
None
Up
Down
E10.5 MPCs scRNA-Seq -Log10 FDR
Log2 fold change(Ptf1aenhΔ/enhΔ/Ptf1a+/+)
Cpa1
Hist1h2ae
Chst2
Clps
Rhov
Cda
Ptf1a
Parm1
Spon1
Gc
P2rx1
Cavin2
Gm266
Ccdc30
Matn4
Kirrel2
Nkx2−2
Mnx1
Tesc
Kcnk3
Fxyd1
Onecut2
Gm10260
Pyy
Hhex
Itih2
Gm11361
Paqr9
Cdh6
Tsix
Sema3c
Steap2
Sptssb
Slc2a3
Enpp2
Nhs
Stxbp5l

Figure S3 - ChIP-Seq profiling of human MPCs and single cell analysis of *Ptf1a*$^{enh\Delta/enh\Delta}$ and *Ptf1a*$^{+/+}$ E10.5 mouse MPCs. Related to Figure 4.

(A) Distribution of H3K27ac, Mediator and PTF1A binding sites in stage 4 derived human MPCs. Proximal sites were defined as 1Kb upstream or downstream of a TSS. **(B)** The majority (91%) of H3K27ac binding sites described in pancreatic MPCs by Cebola *et al*. 2015 were captured in the human MPC H3K27ac dataset from this study. **(C)** Functional term enrichment for genes associated with decreased H3K27ac sites in *PTF1A*$^{enh\Delta/enh\Delta}$ MPCs. **(D,E)** Top sequence motifs at sites with (D) downregulation and (E) upregulation of H3K27ac ($q<0.05$) in *PTF1A*$^{enh\Delta/enh\Delta}$ MPCs. **(F)** PTF1A binding signal at dysregulated H3K27ac sites. Regions that show reduced H3K27ac in *PTF1A*$^{enh\Delta/enh\Delta}$ MPCs are strongly bound by PTF1A. **(G)** Overview of the scATAC-Seq datasets generated from manually dissected E10.5 MPCs (n=5 embryos/genotype). **(H)** Fragment size distribution showing comparable nucleosomal binding patterns in *Ptf1a*$^{enh\Delta/enh\Delta}$ and *Ptf1a*$^{+/+}$ scATAC-Seq datasets (left), as well as enrichments in fragments centered around Ensembl TSS (right). **(I)** Normalized scATAC gene scores of selected marker genes shown on the UMAP projection. **(J)** Differentially accessible peaks between *Ptf1a*$^{enh\Delta/enh\Delta}$ and *Ptf1a*$^{+/+}$ E10.5 MPCs (log2 fold-change ≥ 1.5, binomial test FDR ≤ 0.05). Top motifs enriched in up- and down-regulated peaks are shown. **(K)** Overview of the scRNA-Seq dataset generated from manually dissected E10.5 MPCs (n=9 embryos/genotype). **(L)** QC metrics of E10.5 scRNA-Seq datasets including number of UMIs/gene (left) and number of genes captured per cell (right). **(M)** Normalized expression of selected marker genes shown on 2D projections. **(N)** Volcano plots showing differential single cell gene expression in E10.5 *Ptf1a*$^{enh\Delta/enh\Delta}$ MPCs. Mutant MPCs showed increased expression of some liver-enriched genes such as *Onecut2* and *Hhex*, though not others such as *For1* and *E2f2*, ventral pancreas-enriched genes (*Robo2, Onecut2*, Li et al., 2018) and bile duct epithelium-enriched *Krt17* (see **Table S3**).

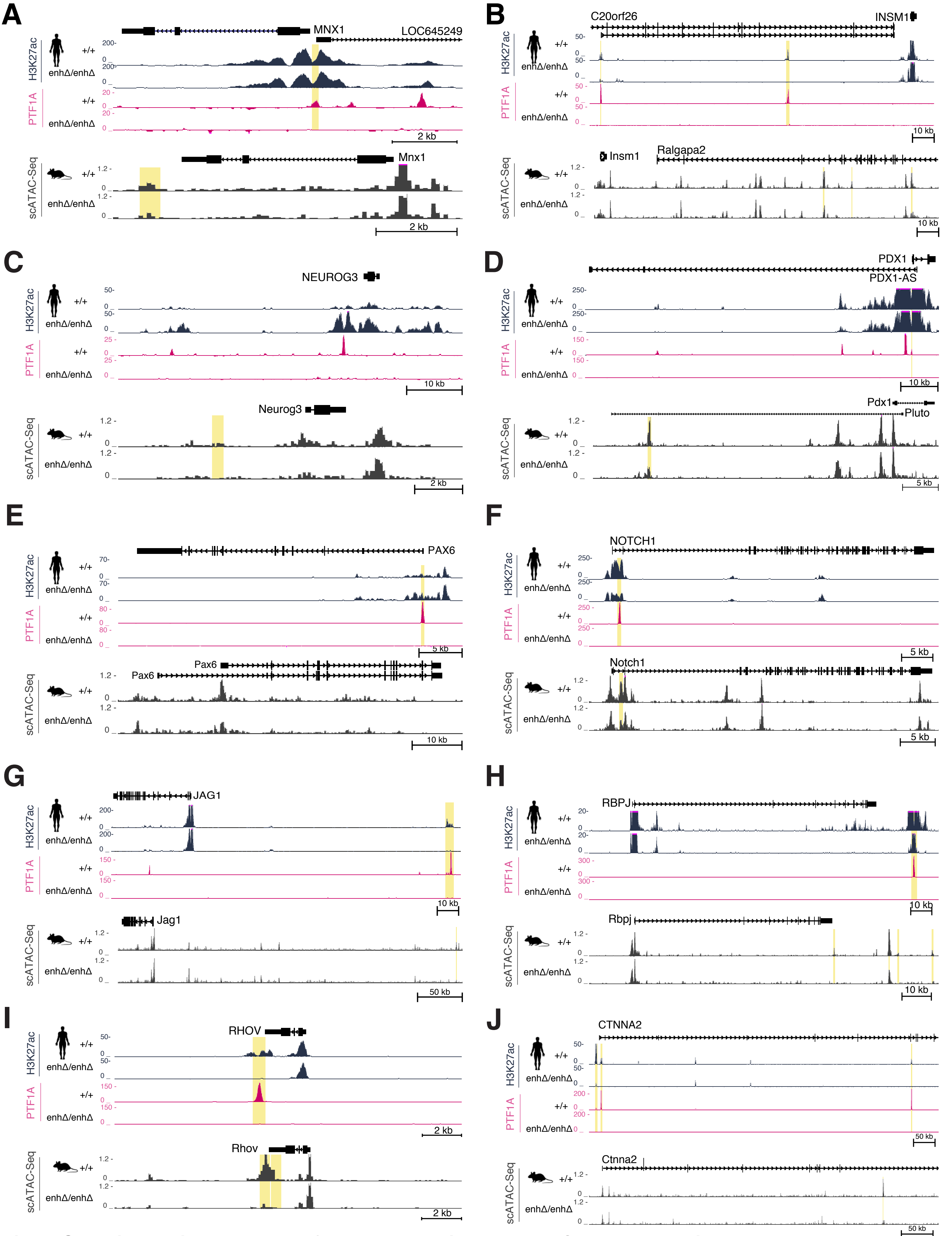

Figure S4. Epigenomic landscapes of PTF1A targets in mutant MPCs. Related to Figure 4.

(A-J) Additional examples of loci showing altered chromatin at PTF1A-bound regions in human mutant cells, and altered chromatin in orthologous or syntenic regions in mouse mutant E10.5 MPCs. Shown are genes that regulate pancreas endocrinogenic transcription factors (*MNX1, INSM1, NEUROG3, PDX1, PAX6*), regulators of Notch signaling (*NOTCH1, RBPJ* and *JAG1*) and tubulogenesis (*RHOV, CTNNA2*). Mouse tracks show aggregated MPC single cell chromatin accessibility.

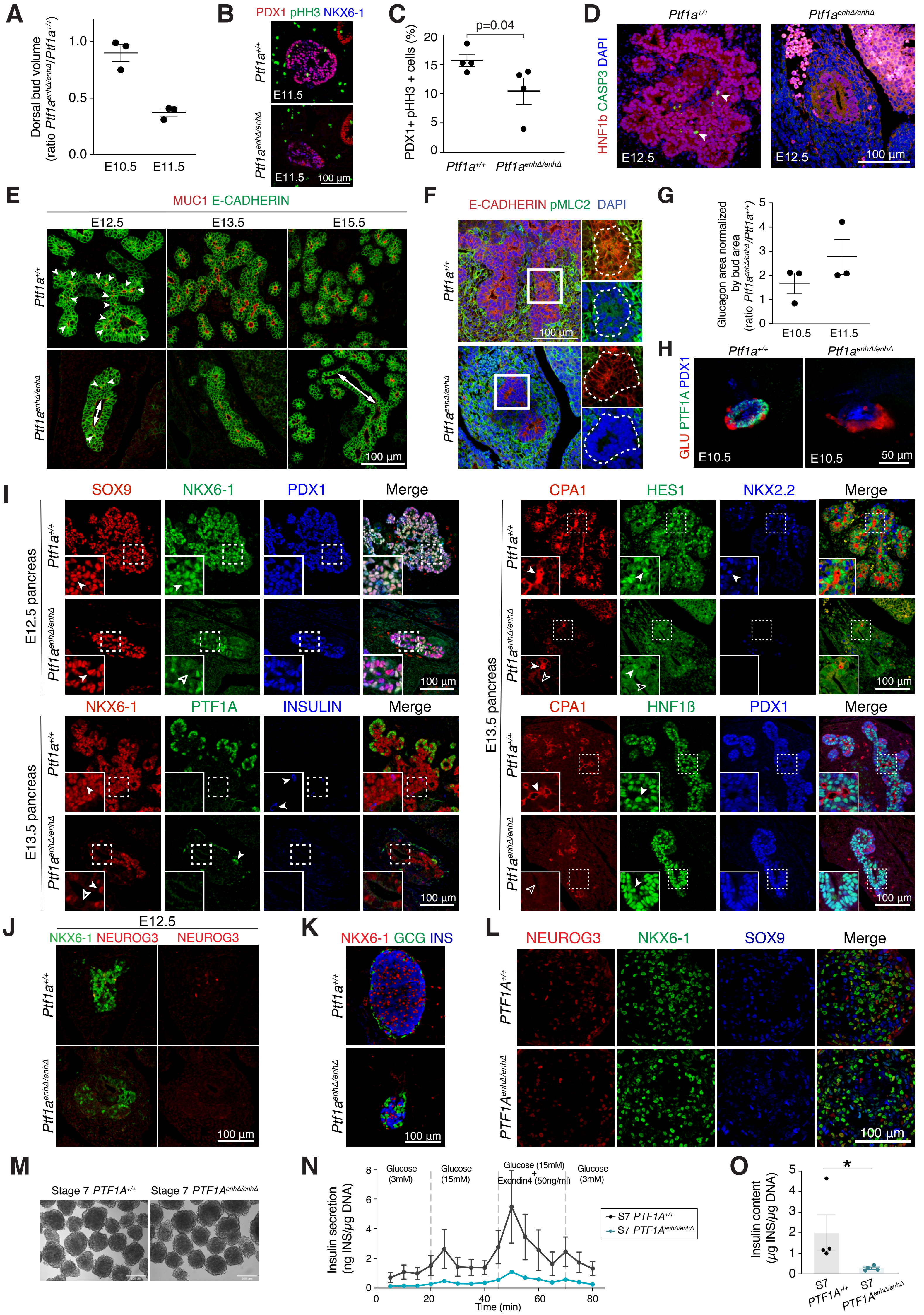

A
Dorsal bud volume (ratio Ptf1a^enhΔ/enhΔ/Ptf1a^+/+)
1.0
0.5
0.0
E10.5
E11.5
B
PDX1 pHH3 NKX6-1
Ptf1a+/+
Ptf1aenhΔ/enhΔ
E11.5
100 µm
C
p=0.04
PDX1+ pHH3 + cells (%)
Ptf1a+/+
Ptf1aenhΔ/enhΔ
D
HNF1b CASP3 DAPI
E12.5
100 µm
E
MUC1 E-CADHERIN
E12.5
E13.5
E15.5
F
E-CADHERIN pMLC2 DAPI
G
Glucagon area normalized by bud area (ratio Ptf1a^enhΔ/enhΔ/Ptf1a^+/+)
H
GLU PTF1A PDX1
E10.5
50 µm
I
SOX9
NKX6-1
PDX1
Merge
E12.5 pancreas
E13.5 pancreas
PTF1A
INSULIN
CPA1
HES1
NKX2.2
HNF1ß
J
E12.5
NKX6-1 NEUROG3
NEUROG3
K
NKX6-1 GCG INS
L
NEUROG3
SOX9
PTF1A+/+
PTF1AenhΔ/enhΔ
M
Stage 7 PTF1A+/+
Stage 7 PTF1AenhΔ/enhΔ
N
Glucose (3mM)
Glucose (15mM)
Glucose (15mM) + Exendin4 (50ng/ml)
Glucose (3mM)
Insulin secretion (ng INS/µg DNA)
Time (min)
S7 PTF1A+/+
S7 PTF1AenhΔ/enhΔ
O
*
Insulin content (µg INS/µg DNA)
S7 PTF1A+/+
S7 PTF1AenhΔ/enhΔ

Figure S5. Defective growth and mophogenesis in *PTF1A*^{enhP} mutants leads to defective trunk cell differentiation. Related to Figure 5.

(A) Dorsal pancreatic bud volumes quantified from *in toto* immunofluorescence, showing decreased size in E10.5 and E11.5 mutant embryos. Volumes are expressed as ratios in *Ptf1a*$^{enhΔ/enhΔ}$ vs. *Ptf1a*$^{+/+}$ embryos at E10.5 or E11.5; n = 3 for each stage and genotype, analyzed with Student's t-test. **(B)** pHH3 immunofluorescence (green) in PDX1+ (red) NKX6-1+ (blue) MPCs from E11.5 *Ptf1a*$^{enhΔ/enhΔ}$ and *Ptf1a*$^{+/+}$ embryos. **(C)** Percentage of PDX1+ MPCs from E10.5 and E11.5 embryos that show proliferative marker pHH3. n = 4 embryos/genotype, analyzed with Student's t-test. **(D)** Caspase3 immunofluorescence analysis showing no increase in apoptosis in mutant E12.5 pancreas. Arrowheads indicate apoptotic cells. **(E)** Mucin1 (red), E-cadherin (green) co-staining show preserved apical Mucin, decreased number of microlumens (arrows), as well as reduced epithelial outgrowth and arborization of the mutant pancreas. **(F)** Loss of apical pospho-myosin light chain 2 (pMLC2, green) in *Ptf1a*$^{enhΔ/enhΔ}$ E12.5 pancreatic epithelial cells. Right panels are zoomed-in images of the squared sections. White dotted lines indicate rosettes around lumen. **(G)** Quantification of first transition endocrine cells in E10.5 and E11.5 *Ptf1a*$^{enhΔ/enhΔ}$ *vs. Ptf1a*$^{+/+}$ embryos showed preserved endocrine cell formation. By E11.5, as the pancreatic mutant growth defect became more apparent, there was a visible increase in the relative area of glucagon+ cells/PDX1+ cells from the dorsal and ventral buds (n=3 embryos/genotype). **(H)** Representative image of an E10.5 *Ptf1a*$^{enhΔ/enhΔ}$ dorsal bud with a relative increase in glucagon+ cells. See also **Video S1** and **Figure 2C**. **(I)** A subset of trunk cells in E12.5 or E13.5 *Ptf1a*$^{enhΔ/enhΔ}$ pancreas lack expression of bipotent progenitor differentiation markers including NKX2-2, NKX6-1, and nuclear HES1, whereas HNF1B expression is maintained, and SOX9 is expressed at moderately reduced levels. Inset panels are zoomed-in images of dotted areas. Solid arrowheads indicate expected expression while empty arrowheads indicate lack of expression. **(J)** E12.5 pancreas from *Ptf1a*$^{enhΔ/enhΔ}$ embryos shows marked reduction of NEUROG3+ endocrine progenitors. **(K)** Large islets were never observed in adult *Ptf1a*$^{enhΔ/enhΔ}$ mice. Illustrative example of a small islet in an adult mutant mouse. **(L)** NEUROG3 expression is reduced in *PTF1A*$^{enhΔ/enhΔ}$ beta cell-like (stage 7) islets. **(M)** Representative brightfield images of *PTF1A*$^{enhΔ/enhΔ}$ and control beta cell-like islets used in perifusion experiments**. (N)** Decreased dynamic insulin secretion response in *PTF1A*$^{enhΔ/enhΔ}$ beta cell-like (stage 7) islets (n = 2 independent experiments, using 2 replicates each). **(O)** Decreased average total insulin content in *PTF1A*$^{enhΔ/enhΔ}$ beta cell-like (stage 7) islets (n = 4).

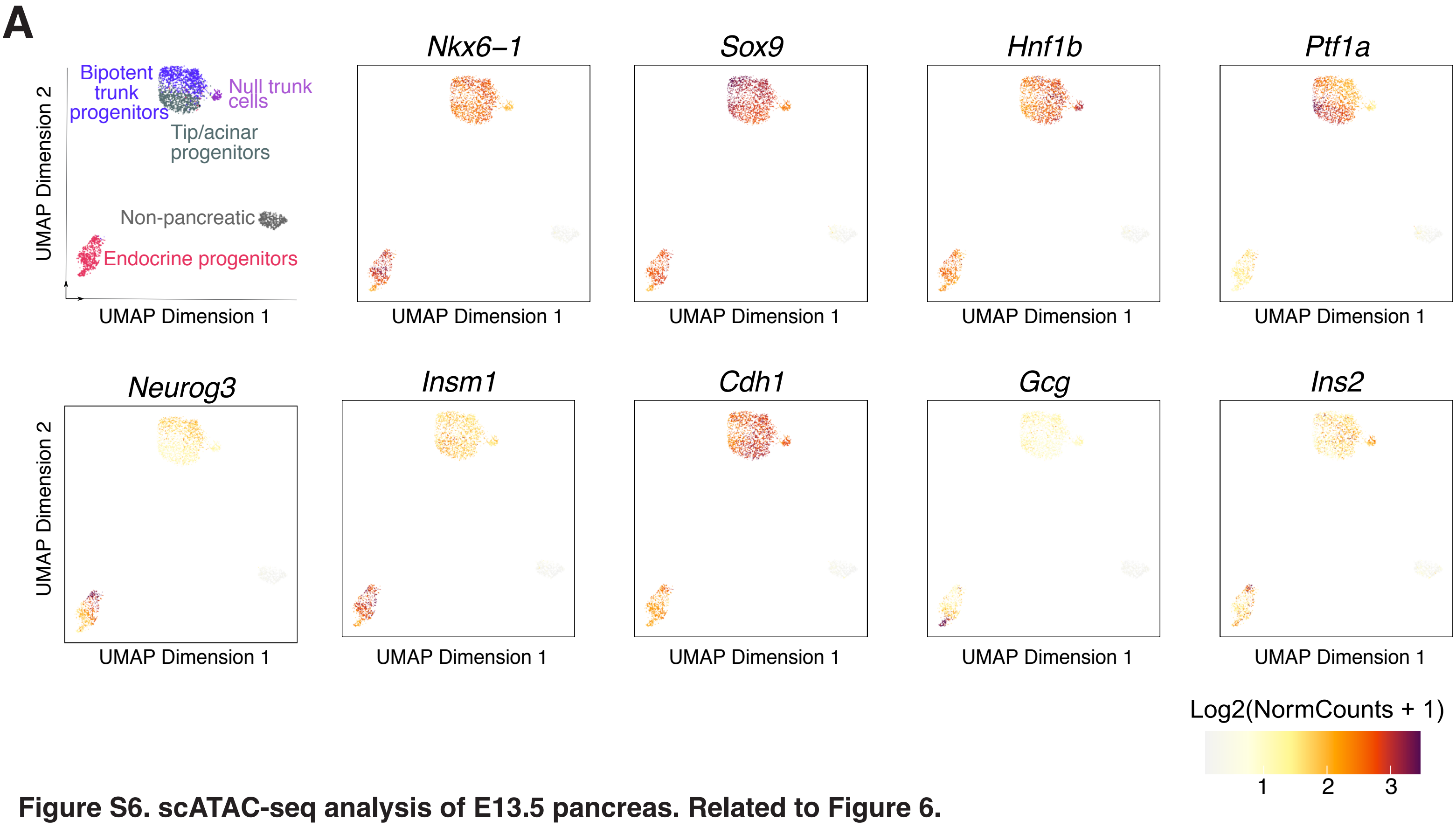

Figure S6. scATAC-seq analysis of E13.5 pancreas. Related to Figure 6.
(A) Normalized gene scores of selected marker genes shown on the UMAP projection of E13.5 scATAC-Seq dataset.

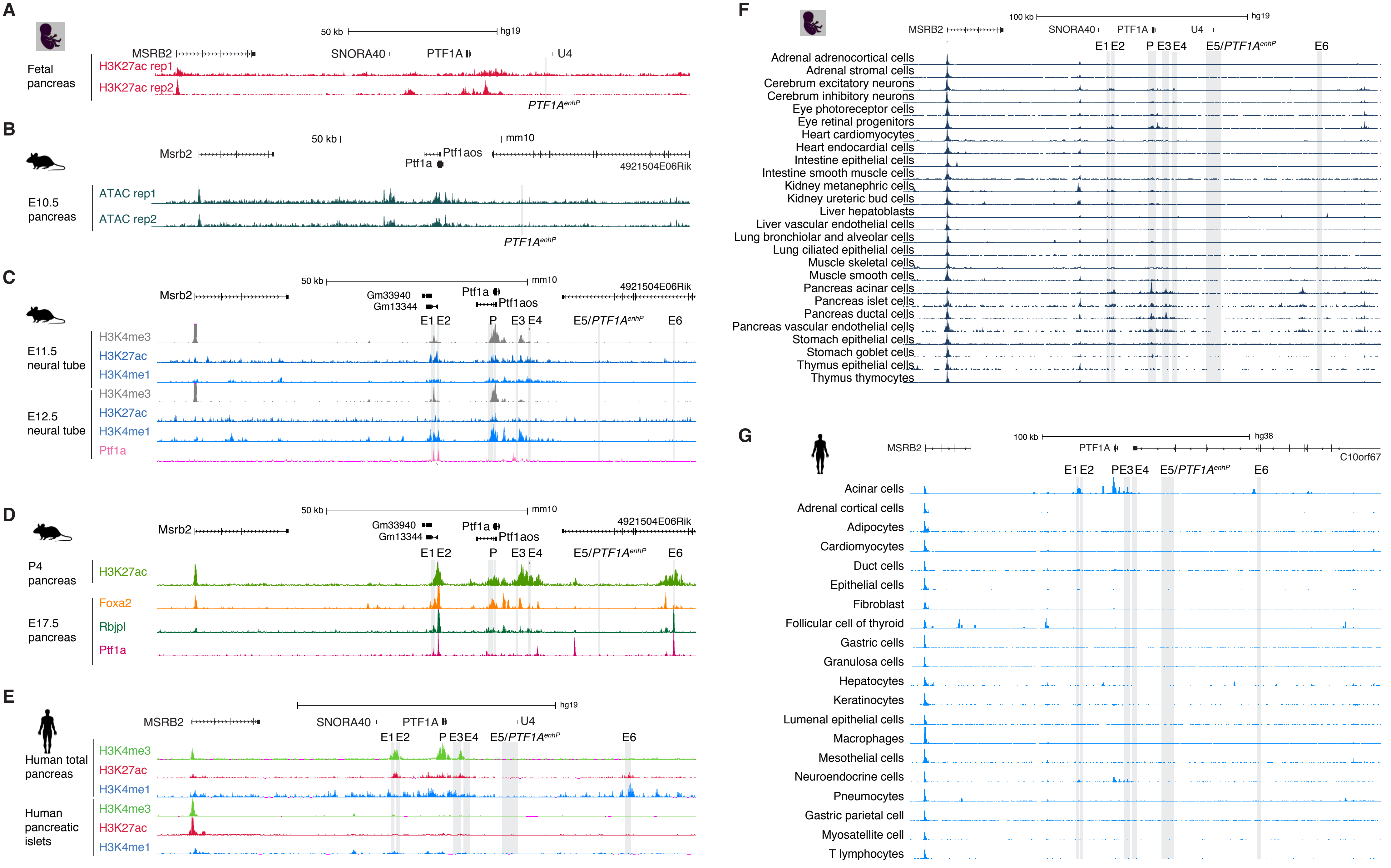

Figure S7. *PTF1A*^{enhP} is specific to MPCs. Related to Figure 7.
(A,B) *PTF1A*^{enhP} is not marked by H3K27ac in chromatin from bulk C19-C21 human fetal pancreas (Gerrard *et al.* 2020), and shows weak accessibility in bulk ATAC-Seq from dissected E10.5 mouse pancreatic buds. **(C)** *PTF1A*^{enhP} is not active in mouse embryonic neural tube. E11.5 and E12.5 histone mark ChIP-Seq datasets are from ENCODE Project Consortium (2020). PTF1A ChIP-Seq in neural tube was re-analyzed from (Meredith *et al.* 2013). Grey highlights point to E1-6 enhancers,as depicted in **Figure 7**. **(D,E)** *PTF1A*^{enhP} is not active in mouse perinatal pancreas or adult human pancreas or pancreatic islets. Mouse pancreas H3K27ac ChIP-Seq datasets were re-analyzed from Kalisz *et al.* 2020 and transcription factor ChIPs are from (Meredith *et al.* 2013). Histone mark ChIP-Seq datasets from total human pancreas and islets were taken from Human Epigenome Consortium (2020) and Miguel-Escalada *et al.*, 2019 respectively. Sequence-depth normalized ChIP signal is shown, where none of the samples have chromatin accessibility in the E5/*PTF1A*^{enhP} region. **(F,G)** *PTF1A*^{enhP} is not accessible in a range of human fetal tissues (Domcke *et al.*, 2020) or adult tissues (Zhang *et al.*, 2021) profiled by scATAC-Seq. Representative cell types from the atlas are shown, where none of the samples have chromatin accessibility in the *PTF1A*^{enhP} region.
